# Unveiling the Chemical Composition of Sulfur-Fumigated Herbs: A Triple Synthesis Approach Using UHPLC-LTQ-Orbitrap MS—A Case Study on Steroidal Saponins in Ophiopogonis Radix

**DOI:** 10.3390/molecules29030702

**Published:** 2024-02-02

**Authors:** Yanan Li, Pingping Dong, Zhanpeng Shang, Long Dai, Shaoping Wang, Jiayu Zhang

**Affiliations:** 1 School of Traditional Chinese Medicine, Binzhou Medical University, Yantai 264003, China; 18353199305@163.com; 2 School of Pharmacy, Binzhou Medical University, Yantai 264003, China; dailong6451@163.com; 3 School of Pharmacy, Shandong University of Traditional Chinese Medicine, Jinan 250355, China; 4 State Key Laboratory for Quality Research of Chinese Medicines, Macau University of Science and Technology, Macao SAR 999078, China; 17864191173@163.com; 5 School of Pharmacy, Beijing University of Chinese Medicine, Beijing 100191, China; zpshang1206@163.com

**Keywords:** Ophiopogonis Radix, sulphur fumigation, UHPLC-LTQ-Orbitrap, steroidal saponins, paired-diagnostic product ion, neutral loss filter

## Abstract

Ophiopogonis Radix (OR) is a traditional Chinese medicine. In recent years, in order to achieve the purpose of drying, bleaching, sterilizing and being antiseptic, improving appearance, and easy storage, people often use sulfur fumigation for its processing. However, changes in the chemical composition of medicinal herbs caused by sulfur fumigation can lead to the transformation and loss of potent substances. Therefore, the development of methods to rapidly reveal the chemical transformation of medicinal herbs induced by sulfur fumigation can guarantee the safe clinical use of medicines. In this study, a combined full scan-parent ions list-dynamic exclusion acquisition-diagnostic product ions analysis strategy based on UHPLC-LTQ-Orbitrap MS was proposed for the analysis of steroidal saponins and their transformed components in sulfur-fumigated Ophiopogonis Radix (SF-OR). Based on precise mass measurements, chromatographic behavior, neutral loss ions, and diagnostic product ions, 286 constituents were screened and identified from SF-OR, including 191 steroidal saponins and 95 sulfur-containing derivatives (sulfates or sulfites). The results indicated that the established strategy was a valuable and effective analytical tool for comprehensively characterizing the material basis of SF-OR, and also provided a basis for potential chemical changes in other sulfur-fumigated herbs.

## 1. Introduction

Traditional Chinese medicine (TCM) needs to be processed before it can be used to treat various diseases. This is one of the characteristics of the clinical use of Chinese medicine. As a unique pre-processing method, sulfur fumigation (SF) is a highly efficient and vital traditional post-harvest handling process for foods, agriculture products, and TCM [1,2]. The mechanism of SF is that sulfur burns at high temperatures to generate SO_2_, which prevents pest infestation, mold, and bacterial contamination and provides a favorable appearance [3]. In a humid environment, SO_2_ combines with water to produce reductive components, which play a role in reducing the coloring components to facilitate the drying of the material [4]. However, residual SO_2_ can induce respiratory symptoms such as cough, chest tightness, and throat irritation [5,6]. Therefore, SO_2_ content is proposed as an official standard requirement for the quality control of sulfur-fumigated medicinal herbs in China [7] and many other countries. In addition, SF can trigger chemical transformations of original bioactive components to generate characteristic sulfate and sulfite derivatives in fumigated herbs [8,9,10]. However, the evaluation of Chinese medicinal materials for SF remains at the level of sulfur dioxide residues (including sulfite derivatives) in the Pharmacopoeia of the People’s Republic of China. This does not truly reflect the transformations in the process of SF. Thus, it is essential to develop a rapid and sensitive approach to ascertain the SF state of a given medicinal herb for TCM quality control.

Traditionally, the detection of common constituents with predictable molecular weights is accomplished by acquiring full-scan LC/MS data followed by the generation of extracted ion chromatograms corresponding to their mass-to-charge (*m*/*z*) values. However, not all constituents, especially microconsitutents, can be detected in full-scan MS data because of their differing amounts and poor chromatographic separation. Their MS/MS acquisitions cannot be triggered when coeluted with the constituents of a relative higher content [11]. Therefore, a new strategy to enhance the constituent detection and identification capacities of LC-MS/MS was established. Since the multiple constituents contained in a specific traditional herb are derived from one or more certain biosynthetic pathways, these constituents could usually be structurally classified into several chemical families with the same carbon skeletons or substructures. So, it is easily understood that their formulas and molecular weights are predictable. Additionally, constituents with the same carbon skeletons will undergo similar fragmentation pathways in collision-induced dissociation (CID) mode, and thus generate similar diagnostic product-ions (DPIs) from their common carbon skeletons. In other words, a series of DPIs representing a specific parent nucleus or substitution groups can be used as the characteristic peaks to select out the corresponding chemical family [12,13,14].

Ophiopogonis Radix (Maidong in Chinese, OR), a widely used TCM published initially in the Herbal Canon of Shen Nong, originates from the dried roots of *Ophiopogon japonicus* (L. f.) Ker-Gawl. According to traditional Chinese medicine theory, OR nourishes the yin, promotes body fluid production, moistens the lung, eases the mind, and clears heart fire [7]. Phytochemical studies have revealed the presence of various biologically active compounds, including steroidal saponins, homoisoflavonoids, and polysaccharides. These compounds have therapeutic effects against acute and chronic inflammation, diabetes, cardiovascular diseases, and other disorders [15]. However, there are limited studies focused on SF-OR. Due to the complexity of the steroidal saponin composition, the full chemical transformation of SF-OR has not been obtained so far. Whether sulfur fumigation alters the chemical composition of OR and the identification of new sulfur-containing derivatives have become important issues for the effectiveness and safety control of OR. Therefore, there is an urgent need to develop a rapid, generally reliable, and accurate analytical method to fully characterize the chemical transformation of OR induced by sulfur fumigation.

In this study, we established a laboratory simulation method to obtain SF-OR samples. Then, a UHPLC-LTQ-Orbitrap MS combined with parent ions list-dynamic exclusion (PIL-DE) acquisition and a DPIs analysis was developed as a strategy for the comprehensive screening and identification of the steroidal saponin constituents of SF-OR. Conversely, a case study was used to validate the effectiveness and feasibility of the proposed strategy. It provides a basis for the identification of other sulfur-fumigated herbal components.

## 2. Results

### 2.1. Identification of Steroidal Saponins from SF-OR

#### 2.1.1. The Establishment of an Analytical Strategy

**Figure 1:**
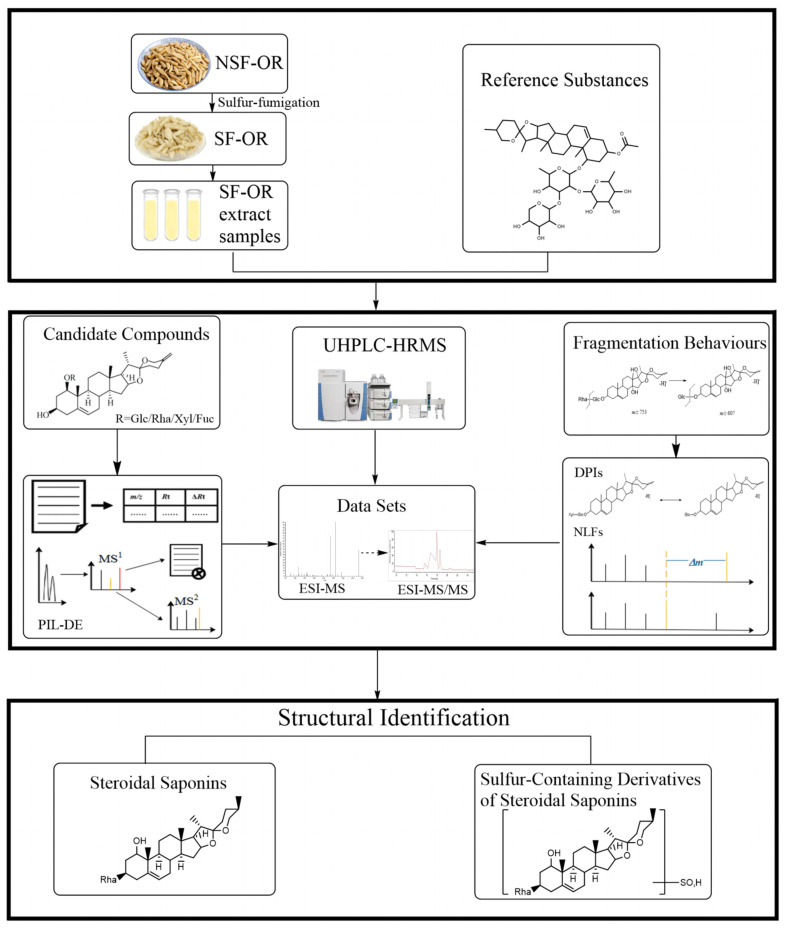
Summary diagram of the developed strategy and methodology.

An efficient and integrated strategy was established for the target identification of steroidal saponins and their sulfur-containing derivatives in SF-OR using a UHPLC-LTQ-Orbitrap MS coupled with post-acquisition data-mining processing techniques (Figure 1).

First, the samples were injected into the UHPLC-LTQ-Orbitrap MS to gain high-resolution mass spectrometry (HRMS) data with full MS scanning acquisition. Second, the reactions (hydroxylation, glucosylation, xylosation, and rhamnosylation) were predicted using information derived from structural characteristics, the literature, and compound databases. A preliminary screening of the candidate compounds was performed using the Thermo Xcalibur 2.1 software to obtain their retention times and accurate molecular weights. According to the PIL-DE scan mode, multi-stage mass spectrometry data were collected. Third, to guide the subsequent rapid analysis, these compounds’ DPI and specific neutral loss filter (NLF) were summarized based on the mass spectrometric cracking rules reported in the literature and the cracking information of reference substances.

#### 2.1.2. Molecular Design of Steroidal Saponins from Ophiopogonis Radix

**Figure 2:**
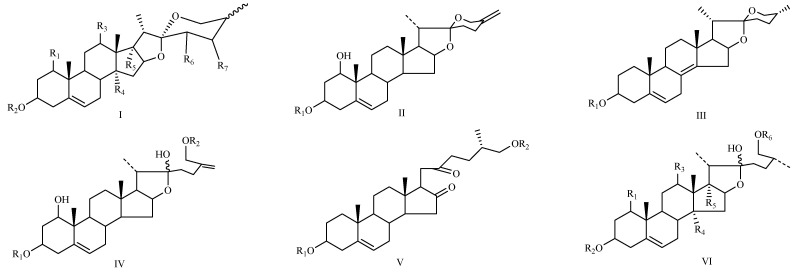
Core structures of six steroidal saponins. Type-I: spirostanol; Type-II and Type-III: deformed spirostanol with a C_5_-C_6_ double bond and a carbohydrate at C_3;_ Type-IV: deformed furostanol with a C5-C6 double bond and two carbohydrates at C3 and C26; Type-V: deformed furostanol with ring opening producing two carbonyls in the E-ring.

The steroidal saponins of OR are usually oligoglycosides of either spirostanol or furostanol (aglycone). According to the different structures of these steroidal saponins, they can be classified into ruscogenin, nitogenin, pennogenin, diosgenin, neoruscogenin, and furostanol saponins, etc. Rhamnose (Rha), fucose (Fuc), glucose (Glc), xylose (Xyl), and arabinose (Ara) are the main monosaccharides present in OR steroidal saponins. Different monosaccharides have unique connections in steroidal saponins [16,17,18,19,20]: (1) Fuc and Glc have priority when connecting steroidal saponins. (2) The monosaccharides in steroidal saponins appear in the following probability sequence: Rha > Fuc > Xyl > Glc > Ara. (3) Spirostanol saponins contain only monosaccharides at C_3_, and the maximum number of sugar units is 3. Arabionose is present only in spirostanol saponins. (4) Furostanol steroidal saponins generally contain two sugars, 1 to 2 Glc at C_26_ and an acetyl group (AC) at C_3_. Therefore, in this study, six steroidal saponins (as shown in Figure 2) were used as the core structure, and Rha (0–2), Fuc (0–2), Xyl (0–2), Glc (0–4), Ara (0–1), and AC (0–1) were used as substituents in the molecular design.

#### 2.1.3. Construction of Ion Lists

**Figure 3:**
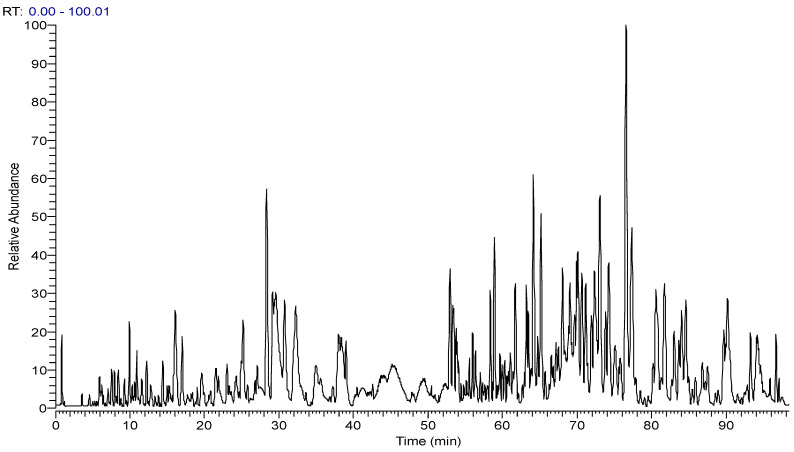
The total ion chromatograms of SF-OR were obtained in full scan mode.

**Figure 4:**
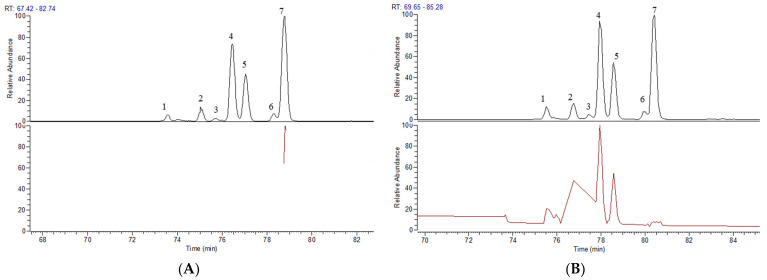
Data acquisition efficiency of compound *m*/*z* 721.4157 in full scan mode (**A**) and PIL-DE mode (**B**).

The Thermo Xcalibur 2.1 software was used to accurately calculate the molecular weights of the above candidate formulas with an error of ±10 ppm. Ion peaks with an intensity greater than 1.0 × 10^4^ in the full scan map (Figure 3) were extracted as potential steroidal saponins of OR. For example, taking compounds with a molecular weight at *m*/*z* 721.4157, seven chromatographic peaks were extracted from the HR-MS^1^. Only the secondary mass spectrum of peak-7 was obtained (Figure 4A), while, in the PIL-DE scan mode, the ESI-MS/MS spectra of these seven peaks were obtained through one data acquisition (Figure 4B). The PIL-DE scan mode dramatically increases the information acquisition efficiency of multi-stage mass spectra.

#### 2.1.4. Analysis of the Characteristic Fragmentation Mechanism of Steroidal Saponins

The DPIs and NLFs were speculated according to the summarized fragmentation behaviors of known compounds, which supplied tremendous help in identifying the secondary metabolites in Ophiopogonis Radix.

##### The Characteristic Fragmentation Mechanism of Type-I Steroidal Saponins

**Figure 5:**
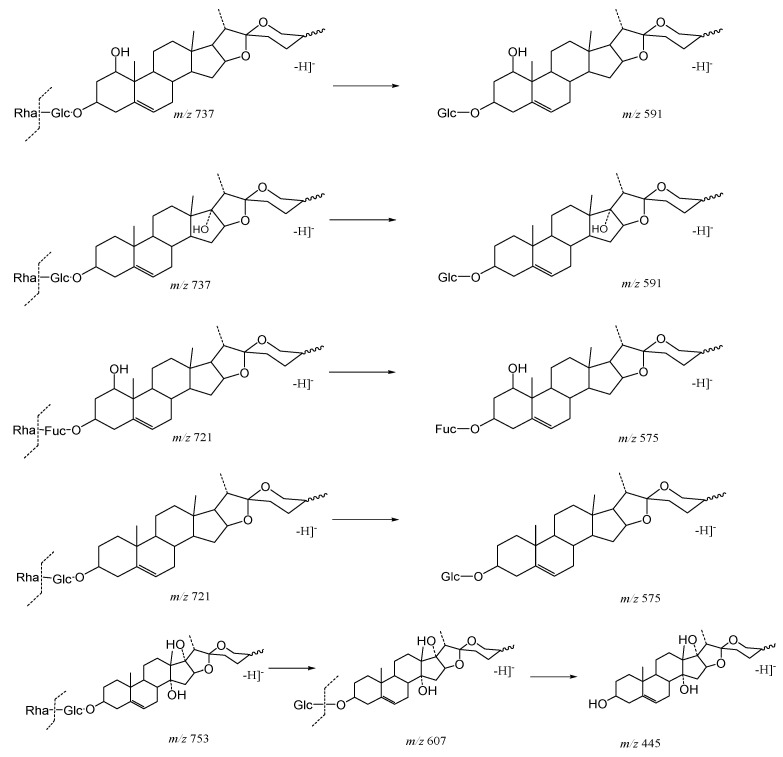
The characteristic fragmentation mechanism of Type-I steroidal Saponins.

**Figure 6:**
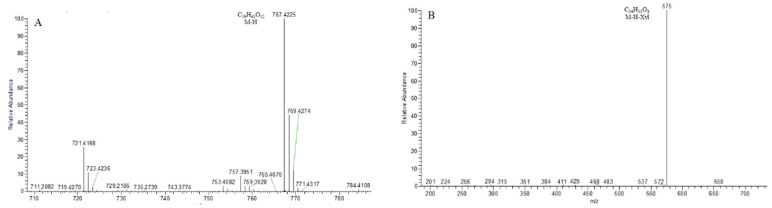
The ESI-MS spectrum (**A**) and ESI-MS/MS spectrum (**B**) of ophiopogonin C′ in negative ion mode.

As shown in Figure 5, the core structure of Type-I steroidal saponins was spirostanol, which generally had only one carbohydrate chain attached to the C_3_ position. In ESI^−^ mode, carbohydrates were removed one by one until a monosaccharide remained on the core structure; hence, most of their aglycon fragment ions were not observed. Take ophiopogonin C′ as an example (Figure 6); it gave rise to a [M − H]^−^ ion at *m*/*z* 721.4157 (C_39_H_61_O_12_, <5 ppm) in negative mode. In its ESI-MS/MS spectrum, the product-ion at *m/z* 575 ([M − H − Rha]^−^) indicated the presence of the Rha group. According to the literature [21,22,23], there was a Glc group in ophiopogonin C′, while NLF of 162 Da was not observed in this experiment. This observation indicated that the Glc group should be directly attached to the core structure, and Rha was attached to the Glc. Based on the above analysis, the molecular formula of the core structure was deduced to be C_27_H_42_O_3_ with a molecular weight of 414 Da. Similarly, ophiopogonin B possessed the same [M − H]^−^ ion at *m*/*z* 721.4170 in the ESI-MS spectrum and a fragment ion at *m*/*z* 575 ([M − H − Fuc]^−^) in the ESI-MS/MS spectrum with ophiopogonin C′ in the negative mode, as shown in Figure S1. The fucose disaccharide was present at C_1_ and the hydroxyl group at C_3_ of ophiopogonin B [23]. Therefore, the molecular formula of the core structure of ophiopogonin B was C_27_H_42_O_4_ with a molecular weight of 430 Da.

**Figure 7:**
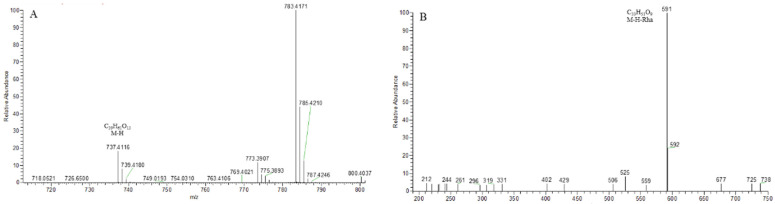
The ESI-MS spectrum (**A**) and ESI-MS/MS spectrum (**B**) of 14-hydroxydiosgenin 3-*O*-α-l-rha-(1→2)-β-d-glc in negative ion mode.

14-hydroxydiosgenin 3-*O*-α-l-rha-(1→2)-β-d-glc showed its [M − H]^−^ ion at *m*/*z* 737.4106 (C_7_H_5_O_3_) with a mass error within 5 ppm. On account of the consecutive neutral loss of Rha and Glc, the fragment ions at *m*/*z* 591 and *m*/*z* 429 were generated in its ESI-MS/MS spectrum, suggesting the presence of a disaccharide chain. Thus, the molecular formula of the core structure was deduced to be C_27_H_42_O_4_ (430 Da), which was consistent with ophiopogonin B and had one more hydroxyl group than that of ophiopogonin C′. The ESI-MS and ESI-MS/MS spectra of 14-hydroxydiosgenin 3-*O*-α-l-rha-(1→2)-β-d-glc are shown in Figure 7. Ophiogenin 3-*O*-α-l-rha-(1→2)-β-d-glc afforded the [M − H]^−^ ion at *m*/*z* 753.4055 (C_39_H_61_O_14_, <5 ppm) in negative ion mode. In the ESI-MS/MS spectrum, it produced fragment ions at *m*/*z* 607 ([M − H − Rha]^−^) and *m*/*z* 445 ([M − H − Rha-Glc]^−^) with consecutive neutral losses of 146 Da (Rha) and 162 Da (Glc) (Figure S2). The molecular formula of the core structure was eventually identified as C_27_H_42_O_5_ (446 Da), which had one more hydroxyl group than that of ophiopogonin B. Due to the polyhydroxy substitution, these compounds generated the fragment ion of the core structure in the ESI^−^ mode, which could be used as a particular fragmentation behavior to provide a reference for subsequent identification.

##### The Characteristic Fragmentation Mechanism of Type-II and Type-III Steroidal Saponins

**Figure 8:**
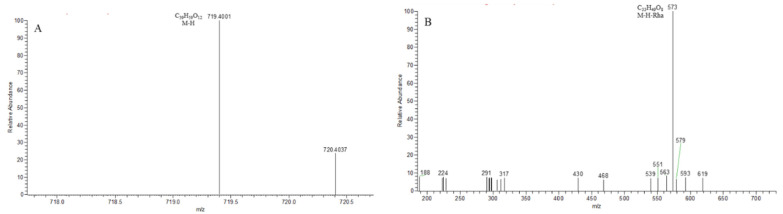
The ESI-MS spectrum (**A**) and ESI-MS/MS spectrum (**B**) of (1β,3β)-3-hydroxyspirost-5,25(27)-dien-1-yl-*O*-6-deoxy-α-l-Rha-(1→2)-β-d-glc in negative ion mode.

The core structures of Type-II and Type-III were deformed spirostanol with a C_5_-C_6_ double bond and a carbohydrate at C_3_. Similarly, most of their aglycon fragment ions were not observed. (1β,3β)-3-hydroxyspirost-5, 25(27)-dien-1-yl-*O*-6-deoxy-α-l-Rha-(1→2)-β-d-glc presented the [M − H]^−^ ion at *m*/*z* 719.4008 (C_39_H_59_O_12_, <5 ppm). In the ESI-MS/MS spectrum, the fragment ion at *m*/*z* 573 ([M − H − Rha]^−^) indicated the presence of the Rha group (Figure 8). According to the literature [24], a Glc group indicated that the Glc group should be directly attached to the core structure, and Rha was attached to the Glc. It was concluded that the molecular formula of the core was C_27_H_40_O_3_ with a molecular weight of 412 Da.

##### The Characteristic Fragmentation Mechanism of Type-IV Steroidal Saponins

**Figure 9:**
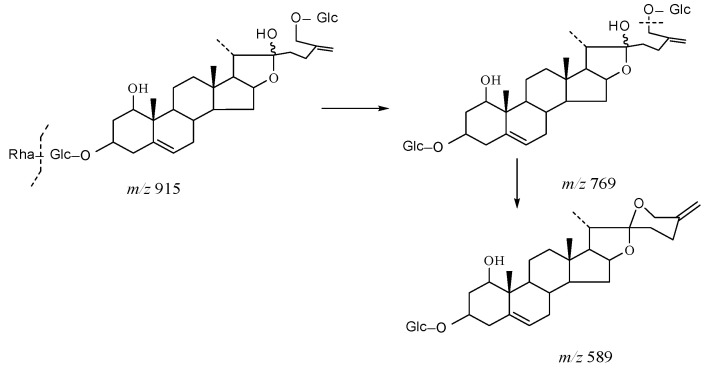
The characteristic fragmentation mechanism of Type-IV steroidal Saponins.

**Figure 10:**
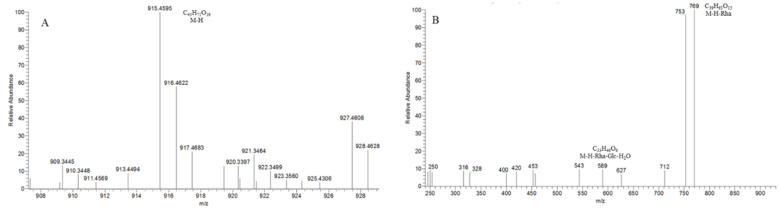
The ESI-MS spectrum (**A**) and ESI-MS/MS spectrum (**B**) of 26-*O*-β-d-Glc-20α-hydroxyfurost-25, 27-dine-3-α-l-Rha-β-d-Glc in negative ion mode.

The core structure of Type-IV was deformed furostanol with a C_5_-C_6_ double bond and two carbohydrates at C_3_ and C_26_ (shown in Figure 9). It is worth noting that the carbohydrate chain at position C_26_ was generally substituted by Glc, which appeared as a specific NLF of 180 Da in the ESI-MS^2^ spectrum. 26-*O*-β-d-Glc-20α-hydroxyfurost-25, 27-dine-3-α-l-Rha-β-d-Glc generated the [M − H]^−^ ion at *m*/*z* 915.4584 (C_45_H_71_O_19_, <5 ppm). In the ESI-MS/MS spectrum, characteristic fragment ions at *m*/*z* 769 ([M − H − Rha]^−^) and *m*/*z* 589 ([M − H − Rha-180]^−^) were observed, which could be conducted as paired-DPI (*p*DPI) for these types of compounds (Figure 10).

##### The Characteristic Fragmentation Mechanism of Type-V Steroidal Saponins

**Figure 11:**
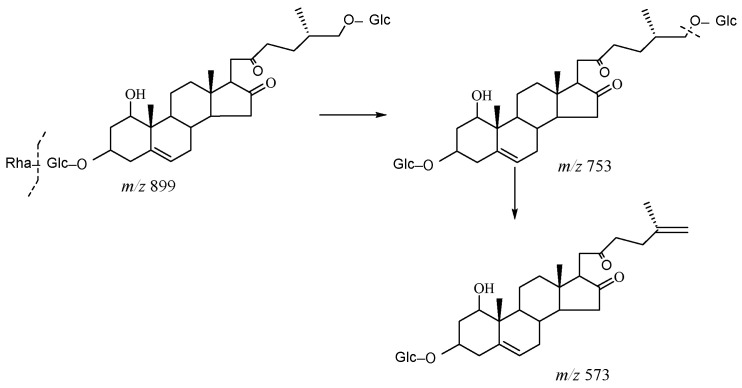
The characteristic fragmentation mechanism of Type-V steroidal Saponins.

As shown in Figure 11, the core structure of Type-V was deformed furostanol with a ring opening producing two carbonyls in the E-ring. There were generally two carbohydrates at C_3_ and C_26_; the carbohydrate chain at position C_26_ was generally substituted by Glc, which appeared as a specific NLF of 180 Da in the ESI-MS^2^ spectrum. For example, (20*R*,25*R*)-26-*O*-β-d-glc-3β, 26-dihydroxycholest-5-en-16, 22-dioxo-3-*O*-α- L-rha(1→2)-β-d-Glc possessed the [M − H]^−^ ion at *m*/*z* 899.4634 (C_45_H_71_O_18_, mass error within 5 ppm). It also produced ions at *m*/*z* 753 ([M − H − Rha]^−^) and *m/z* 573 ([M − H − Rha-180]^−^), suggesting the presence of Type-V *p*DPIs (Figure S3).

##### The Characteristic Fragmentation Mechanism of Type-VI Steroidal Saponins

**Figure 12:**
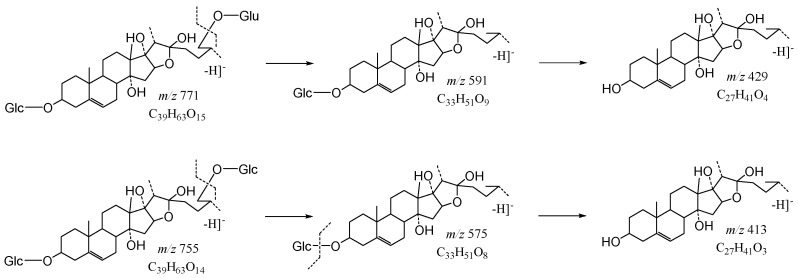
The characteristic fragmentation mechanism of Type-VI steroidal Saponins.

The core structure of Type-VI was furostanol with two carbohydrate chains at C_3_ and C_26_. In negative mode, the carbohydrates were removed one by one until no monosaccharides remained on the core structure; hence, their aglycon fragment ions could be observed. The carbohydrate chain at C_26_ generally contained 1–2 Glc, which would appear as a specific NLF of 180 Da in MS^2^ spectra. The characteristic fragmentation mechanism of Type-V steroidal Saponins is shown in Figure 12. Ophiofurspiside M generated the [M − H]^−^ ion at *m*/*z* 917.4741, with the molecular formula of C_45_H_73_O_19_ and a mass error within 5 ppm. In the ESI-MS^2^ spectrum, a battery of fragment ions at *m*/*z* 771 ([M − H − Rha]^−^), *m*/*z* 591 ([M − H − Rha-180]^−^), and *m*/*z* 591 ([M − H − Rha-180-Glc]^−^) were all observed (Figure S4). Xyl-Ophiofurspiside M, with a mass error within 5 ppm, gave rise to the accurate [M − H]^−^ ion at *m*/*z* 1049.5163 (C_50_H_81_O_23_). Fragment ions at *m*/*z* 917 ([M − H-Xyl]^−^), *m*/*z* 771 ([M − H-Xyl-Rha]^−^), *m*/*z* 591 ([M − H-Xyl-Rha-180]^−^), and *m*/*z* 429 ([M − H-Xyl-Rha-180-Glc]^−^) were generated in the ESI-MS/MS spectrum, which was formed by the consecutive loss of Xyl, Rha, Glc, and Glc (Figure S5). Ophiofurspiside A, with the [M − H]^−^ ion at *m*/*z* 1033.5214 (C_50_H_81_O_22_, <5 ppm), presented fragment ions at *m*/*z* 901 ([M − H-Xyl]^−^), *m*/*z* 755 ([M − H-Xyl-Rha]^−^), and *m*/*z* 575 ([M − H − Rha-180]^−^) (Figure S6). In addition, ophiopogonin H possessed the [M − H]^−^ ion at *m*/*z* 1063.5319 (C_51_H_83_O_23_, mass error < 5 ppm). It generated ESI-MS^2^ DPIs at *m*/*z* 901 ([M − H-Glc]^−^), *m*/*z* 755 ([M − H-Glc-Rha]^−^), and *m*/*z* 575 ([M − H-Xyl-Rha-180]^−^) (Figure S7). Therefore, the *p*DPIs at *m*/*z* 771/591 and *m*/*z* 755/575 could be used to identify Type-VI rapidly.

#### 2.1.5. Determination and Verification of NLFs and DPIs of OR Steroidal Saponins

The different core structures of the six types of steroidal saponins, combined with the relevant information reported in the literature [15], allow the fragmentation mechanism of these compounds in negative ion mode to be analyzed and the NLFs and DPIs to be summarized. The potential NLFs of the OR Steroidal Saponins are summarised in Table 1.

Congeneric compounds generally have a similar MS fragmentation regularity, thereby generating characteristic DPIs that can represent the structure of such compounds. However, it is difficult to accurately identify the structure of natural compounds with a large molecular weight and relatively complex structures based on one DPI. Therefore, in this experiment, the concept of *p*DPI was proposed to provide meaningful guidance for the rapid identification of OR steroidal saponins. According to the components identified and reported in the literature, the *p*DPIs of the six types of core structures are summarized, as shown in Table 2. A total of 9 *p*DPIs of Type-I, 2 *p*DPIs of Type-II and Type-III, 1 *p*DPI of Type IV, 1 *p*DPI of Type-V, and 3 *p*DPIs of Type-VI were found.

#### 2.1.6. Detection and Structural Elucidation of OR Steroidal Saponins

Based on the established *p*DPIs and NLFs, 191 steroidal saponins were quickly identified from OR. There were 105 Type-I, 12 Type-II and Type-III, 13 Type-IV, 18 Type-V, and 42 Type-VI OR steroidal saponin compounds screened and identified. The detailed MS data information is shown in Table 3.

Taking the *p*DPI at *m*/*z* 707/575 of Type-I as an example, Sprengerinin A with the [M − H]^−^ ion at *m*/*z* 707.4011 (C_38_H_59_O_12_, mass error within 5 ppm) generated the DPI at *m*/*z* 575 ([M − H-Xyl]^−^). The NLF of 132 Da (*m*/*z* 707→*m*/*z* 575) indicated the presence of Xyl. The molecular formula corresponding to *m*/*z* 575 was inferred to be C_33_H_51_O_8_ ([Aglycon-H + Glc]^−^/[Aglycon-H + Rha]^−^). In addition, compound **S39** generated its [M − H]^−^ ion at *m*/*z* 839.4423; its molecular formula was deduced to be C_43_H_67_O_16_. In its ESI-MS/MS spectrum, the DPI at *m*/*z* 707 ([M − H-Xyl]^−^) certified the loss of Xyl from the [M − H]^−^ ion. Further, the DPI at *m*/*z* 561 (C_32_H_49_O_8_, [Ruscogenin-H + Ara]^−^) was formed due to the neutral loss of 146 Da from the DPI at *m*/*z* 707. Therefore, S39 was tentatively identified as Ruscogenin 1-*O*-α-l-Xyl (1→3) Rha (1→2) Ara, and the *p*DPI at *m*/*z* 707/561 was initially assigned as Ruscogenin-Rha (1→2) Ara.

S109–S112 possessed identical [M − H]^−^ ions at *m*/*z* 851.4418 (C_44_H_67_O_16_). The DPIs at *m*/*z* 719 ([M − H-Xyl]^−^) and *m*/*z* 573 ([M − H-Xyl-Rha]^−^) were generated in their ESI-MS/MS spectra, suggesting the presence of Xyl and Rha. The molecular formula corresponding to *m*/*z* 573 was inferred to be C_33_H_51_O_8_ ([Aglycon-H + Glc]^−^/[Aglycon-H + Rha]^−^). The core structures of these two aglycones differed by one hydroxyl substitution. Thus, S109–S112 were tentatively characterised as (1β,3β)-3-hydroxyspirosta-5,25(27)-dien-1-yl-*O*-6-deoxy-α-l-Rha-(1→2)-*O*-[β-d-Xyl-(1→4)]-β-d-Fuc or 25(R)-spirost-5, 8-diene-3β-ol-3-*O*-α-l-Rha(1→2)-β-d-Xyl(1→4)-β-d-Glc. The *p*DPI at *m*/*z* 719/573 was used for the preliminary identification of Type-II and Type-III compounds, which was one less hydroxyl than the *p*DPI at *m*/*z* 735/589.

S118 produced the [M − H]^-^ ion at *m/z* 915.4584 (C_45_H_71_O_19_) in the negative ion mode. The *p*DPIs at *m*/*z* 769 ([M − H − Rha]^−^)/589 ([M − H − Rha-180]^−^) were, respectively, generated in its ESI-MS^2^ spectrum. The neutral loss of 180 Da indicated the existence of Glc in the carbohydrate chain at the position of C_26_. The corresponding molecular formula of *m*/*z* 589 was C_33_H_49_O_9_, which was conjectured to be [Aglycon-H + Glc]^−^. Since the neutral loss of Glc was not observed in the ESI-MS/MS spectrum, it was speculated that the other Glc was at C_3_. Thus, S118 could be deduced as 26-*O*-β-d-Glc-20α-hydroxyfurost-25,27-dine-3-α-l-Rha-β-d-Glc or its isomer. The structure represented by the *p*DPI at *m*/*z* 769/569 of Type-IV is shown in Table 2.

S131 afforded the [M − H]^−^ ion at *m*/*z* 899.4634 (C_45_H_71_O_18_). In the MS^2^ spectrum, the DPI at *m*/*z* 753 ([M − H − Rha]^−^) indicated the presence of Rha, and the DPI at *m*/*z* 573 ([M − H − Rha-180]^−^) indicated the presence of Glc at C26 of Rha. The corresponding molecular formula of *m*/*z* 573 was C_33_H_49_O_8_ ([Aglycon-H + Glc]^−^). Since the neutral loss of 162 Da or 180 Da was not observed in the ESI-MS/MS spectrum, it was speculated that the other Glc was directly attached to the core. Therefore, S131 was tentatively identified as (20*R*,25*R*)-26-*O*-β-d-Glc-3β,26-dihydroxycholest-5-en-16,22-dioxo-3-*O*-α-l-Rha(1→2)-β-d-Glc or its isomer. The structure represented by the *p*DPI at *m*/*z* 753/573 of Type-V is shown in Table 2.

S149–S150 showed their theoretical deprotonated molecular ions at *m*/*z* 901.4791 (C_45_H_73_O_18_, mass error within ±5.00 ppm). The *p*DPI of Type-VI at *m*/*z* 755 ([M − H − Rha]^−^)/*m*/*z* 575 ([M − H − Rha-180]^−^) indicated the presence of Glc and Rha substitution at C_26_. The corresponding molecular formula of *m*/*z* 575 was C_33_H_51_O_8_, which was conjectured to be [Aglycone-H + Glc]^−^. Based on the above analysis, the structures of compounds S149–S150 were initially identified. The structure represented by the *p*DPI at *m*/*z* 755/575 was shown in Table 2. S151–S153 possessed the theoretical [M − H]^−^ ions at *m*/*z* 917.4740 (C_45_H_73_O_19_, mass error within ±5.00 ppm). Another *p*DPI of Type-VI at *m*/*z* 771 ([M − H − Rha]^−^)/*m*/*z* 591 ([M − H − Rha-180]^−^) was formed by the neutral loss of Rha and Glc in their ESI-MS/MS spectra. The molecular formula of *m*/*z* 591 was C_33_H_51_O_9_, which should be [Aglycon-H + Glc]^−^. Compared with the *p*DPI at *m*/*z* 755/575, the *p*DPI at *m*/*z* 771/591 had more than one hydroxyl substitution. In addition, S154–S156 provided theoretical [M − H]^−^ ions at *m*/*z* 933.4689 (C_45_H_73_O_20_, mass error within ±5.00 ppm). In the ESI-MS^2^ spectra, the DPIs at *m*/*z* 787 ([M − H − Rha]^−^) and 607 ([M − H − Rha-180]^−^) indicated the core structure was furostanol, and Glc was present at C_26_. The *p*DPI at *m*/*z* 771/591 could also be applied to identify Type-VI steroidal saponins rapidly.

### 2.2. Identification of Sulfur-Containing Derivatives of Steroidal Saponins from SF-OR

#### 2.2.1. Molecular Design of Sulfur-Containing Derivatives of Steroidal Saponins

The reported pathways of ginsenosides during sulfur fumigation are sulfation and sulfite [25]. In addition, sulfur generates SO_2_ at high temperatures to lower the pH, making the glycosides easily hydrolyzed. Therefore, to fully characterize the sulfur-containing derivatives of steroidal saponins in OR, six types of steroidal saponins were used as the core structure, and Rha (0–2), Fuc (0–2), Xyl (0–2), Glc (0–4), Ara (0–1), Ac (0–2), SO_2_ (0–1), and SO_3_ (0–1) were used as substituents for molecular design.

#### 2.2.2. Screening of the Candidate Molecular Weight of Sulfur-Containing Derivatives of Steroidal Saponins

The accurate [M − H]^−^ of the candidate molecular formula was calculated in Section 2.2.1, the chromatographic peak from the ESI-MS spectra was extracted, and the peaks with an intensity > 1.0 × 10^4^ were selected as the potential sulfur-containing derivatives of Steroidal Saponins. According to the established PIL-DE scan mode, MS^2^ data collection was performed.

#### 2.2.3. Identification of Sulfur-Containing Derivatives of Steroidal Saponins

Based on the established structure identification strategy with *p*DPIs and NLFs, rapid screening of the steroidal saponins’ sulfur-containing derivatives was carried out. In addition, the isotope peak [M − H+2]^−^ of the sulfur-containing derivatives under ultra-high resolution split into two peaks with a mass difference of 0.01 Da. Therefore, ^12^C_x_^1^H_y_^16^O_z_^32^S^13^C_2_^18^O and ^12^C_x+2_^1^H_y_^16^O_z+1_^34^S were separated, which could provide a basis for the further confirmation of sulfur-containing derivatives [26].

##### Identification of Type-I Sulfur-Containing Derivatives of Steroidal Saponins

**Figure 13:**
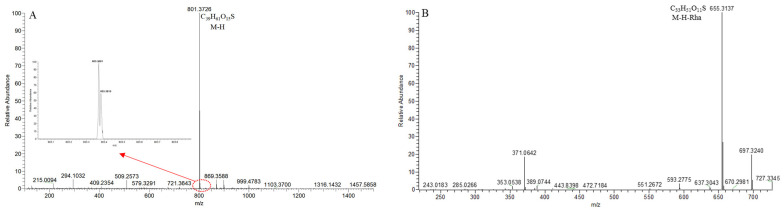
The ESI-MS spectrum (**A**) and ESI-MS/MS spectrum (**B**) of SS9 in negative ion mode.

**SS9**, with the [M − H]^−^ ion at *m*/*z* 801.3714 (C_39_H_61_O_15_S, <5 ppm), was 79.95 Da (SO_3_) more than that of **S2**–**S8**. In the 100,000 FWHM @ 400 *m*/*z* ultra-high resolution mode, its isotope peak at [M − H+2]^−^ formed two peaks at *m*/*z* 803.3691 and *m*/*z* 803.3810, further confirming that **SS9** was a sulfur compound. In its ESI-MS/MS spectrum, the DPI at *m*/*z* 655 ([M − H − Rha]^−^) was consistent with **S2**–**S8** (Figure 13). The *p*DPI it generated at *m*/*z* 801/655 was 79.95 Da more than that of *m*/*z* 721/575 of **S2**–**S8**. Therefore, the compound **SS9** was identified as the sulfated product of the compound **S2**–**S8**.

As shown in Figure S8, **SS10**–**SS14** displayed the [M − H]^−^ ion at *m*/*z* 817.3663 (C_39_H_61_O_16_S, 5 ppm). This was 79.95 Da greater than **S11**–**S15** in negative ion mode, which implied that **SS10**–**SS14** might be the sulfated product of **S11**–**S15**. Two isotope peaks at *m*/*z* 819.3615 and *m*/*z* 819.3772 further indicated that **SS10**–**SS14** were sulfur-containing compounds. The DPI at *m*/*z* 671 ([M − H − Rha]^−^) was consistent with **S11**–**S15**. The *p*DPI at *m*/*z* 817/671 was 79.95 Da greater than the *p*DPI at *m*/*z* 721/575 of **S11**–**S15**. Therefore, the compounds **SS10**–**SS14** were presumed to be the sulfated products of **S11**–**S15**.

According to the characteristic fragmentation mechanism of Type-I steroidal saponins and the specificity of the sulfur-containing compound isotope peaks, a total of 24 Type-I sulfur-containing derivatives of steroidal saponins were identified from SF-OR. They were all sulfated products, as shown in Table 4.

##### Identification of Type-II and Type-III Sulfur-Containing Derivatives of Steroidal Saponins

**Figure 14:**
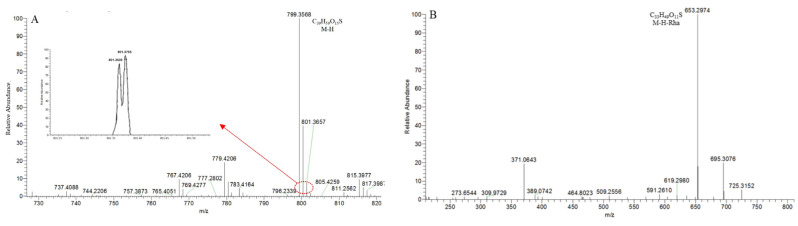
The ESI-MS spectrum (**A**) and ESI-MS/MS spectrum (**B**) of SS30 in negative ion mode.

**SS30,** with the [M − H]^−^ ion at *m*/*z* 799.3563 (C_39_H_59_O_15_S), was 79.95 Da greater than **S106** (Figure 14A). In the 100,000 FWHM @ 400 *m*/*z* ultra-high resolution mode, its isotope peak at [M − H+2]^−^ showed two peaks at *m*/*z* 803.3691 and *m*/*z* 803.3810 due to the existence of element “S”. In the MS^2^ spectrum (Figure 14B), the DPI at *m*/*z* 653 ([M − H-146]^−^) indicated the presence of a Rha or a Fuc. The *p*DPI at *m*/*z* 799/653 of **SS30** was 79.95 Da more than the *p*DPI at *m*/*z* 719/573 of S106, indicating that these two compounds had the exact fragmentation mechanism. Therefore, **SS30** was identified as the sulfated product of **S106**.

According to the characteristic fragmentation mechanism of Type-II and Type-III steroidal saponins and the specificity of the sulfur-containing compound isotope peaks, a total of three Type-II and Type-III sulfur-containing derivatives of steroidal saponins were identified and targeted from SF-OR. They were all sulfated products, as shown in Table 4.

##### Identification of Type-IV Sulfur-Containing Derivatives of Steroidal Saponins

There were no Type-IV sulfur-containing derivatives of steroidal saponins detected in SF-OR.

##### Identification of Type-V Sulfur-Containing Derivatives of Steroidal Saponins

**Figure 15:**
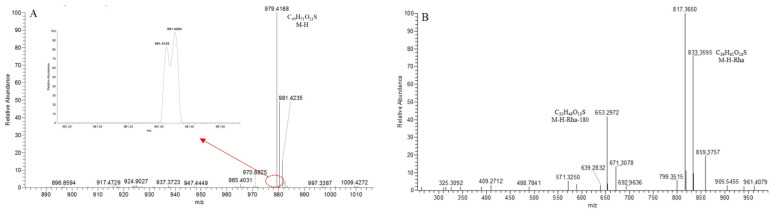
The ESI-MS spectrum (**A**) and ESI-MS/MS spectrum (**B**) of SS40 in negative ion mode.

**SS40** provided the [M − H]^−^ ion at *m*/*z* 979.4192 (C_45_H_71_O_21_S) in the ESI-MS spectrum (Figure 15A). It was 79.95 Da greater than **S131**, indicating that it probably was the sulfated product of **S131**. Two isotope peaks at *m*/*z* 981.4125 and *m*/*z* 981.4264 proved that element “S” existed. The *p*DPI at *m*/*z* 817 ([M − H-146]^−^)/*m*/*z* 637 ([M − H-146-180]^−^) was 79.95 Da more than that of **S106** (Figure 15B), indicating that **SS40** was the sulfonation product of **S106**.

Based on the mass characteristic fragmentation mechanism of Type-V steroidal saponins, a total of eight Type-V steroidal saponins’ sulfur derivatives were identified from the targeted screening of OR, all of which belonged to sulfated products, as shown in Table 4.

##### Identification of Type-VI Sulfur-Containing Derivatives of Steroidal Saponins

**Figure 16:**
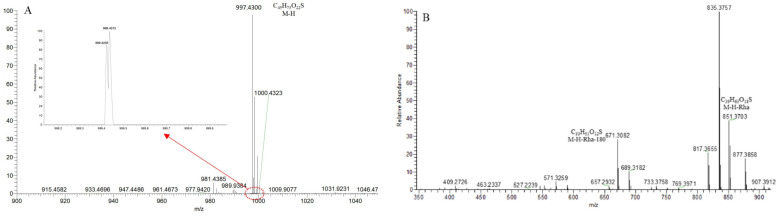
The ESI-MS spectrum (**A**) and **ESI**-MS/MS spectrum (**B**) of SS61 in negative ion mode.

**SS61**, with the [M − H]^−^ ion at *m*/*z* 997.4303 (C_45_H_73_O_22_S), was 79.95 Da (SO_3_) greater than Ohiopojaponin B and showed two isotope peaks at *m*/*z* 999.4235 and *m*/*z* 999.4373 (Figure 16A). In the ESI-MS^2^ spectrum (Figure 16B), the *p*DPIs at *m*/*z* 851 ([M − H-146]^−^)/*m*/*z* 671 ([M − H-146-180]^−^) were, respectively, attributed to the neutral losses of Rha and Glc. The core structure was inferred as furostanol or pseudo-furostanol. The *p*DPI at *m*/*z* 851/671 was 79.95 Da more than *m*/*z* 771/591 of **S106**, confirming that **SS61** and Ohiopojaponin B had the exact fragmentation mechanism. Ultimately, **SS61** was identified as the sulfated product of Ohiopojaponin B.

For compound **SS72**–**SS74**, the ESI^-^ fragment ion with *m*/*z* 1127.4726 was designated as the quasi-molecular ion [M − H]^−^, which indicated that the possible elemental composition was C_51_H_83_O_25_S. In the 100,000 FWHM @ 400 *m*/*z* ultra-high resolution mode, two isotope peaks at *m*/*z* 1129.4956 and *m*/*z* 1129.5038 split from the ion [M − H+2]^−^ of element “S”, and DPIs at *m*/*z* 997 ([M − H − Rha]^−^), *m*/*z* 981 ([M − H-Glc]^−^), *m*/*z* 917 ([M − H-Glc-SO_2_]^−^), and *m*/*z* 801 ([M − H-Glc-180]^−^) were observed (Figure S9), meaning that their fragmentation mechanism was consistent with that of Trigoneoside IVa. Therefore, **SS72**–**SS74** was eventually identified as Trigoneoside IVa sulfite.

A total of 55 Type-VI steroidal saponins’ sulfur derivatives were identified from the targeted screening of OR based on the fragmentation mechanism of Type-VI steroidal saponins, in which 10 compounds were sulfite products and 45 were sulfate products, as shown in Table 4.

## 3. Discussion

UHPLC-LTQ-Orbitrap has become a powerful tool for drug analysis with its high sensitivity, accuracy, and separation ability [27]. However, the spectral information contained in existing chemical standards and databases is minimal. The LC-MS technique alone cannot satisfy the structural characterization of complex and diverse TCM [28]. Therefore, more new techniques and research strategies are needed to meet this challenge. Based on this, the study proposes a combined full scan-parent ions list-dynamic exclusion acquisition-diagnostic product ions analytical strategy for revealing the chemical composition of sulfur-fumigated TCM. The core technique of the strategy is to predict the possible molecular structures based on the structural characteristics of the compounds. A UHPLC-LTQ-Orbitrap high-resolution mass spectrometer is then utilized for data acquisition. After the initial screening of candidate compounds, multi-stage mass spectrometry data are targeted based on the PIL-DE scanning mode [12]. By studying the mass spectral cleavage behavior of the representative compounds, the characteristic cleavage patterns of compounds with different parent nucleus structures are summarized, and the DPIs and NLFs of such compounds are inferred. In addition, for natural products with a high molecular weight and relatively complex structures, it is often difficult to accurately characterize their structures based on one DPI [29]. Therefore, the concept of *p*DPI is proposed in the strategy. Accordingly, the rapid identification of chemical components is performed.

The proposed strategy was extensively investigated for the steroidal saponins of SF-OR. The successful implementation of the strategy accelerated the identification of the steroidal saponin components, expanded the search scope, and ensured the accuracy of the component identification. Although the strategy has many advantages, there are still some limitations. First, complex matrices and impurities in the samples may interfere with the analytical results, affecting accuracy and sensitivity. Second, the parent ion scanning technique has certain requirements for the sample concentration, as too high or too low will affect the detection sensitivity. Nevertheless, this innovative measure has broad application value in scientific research and medical diagnosis, while also providing a basis for the identification of compounds and their pharmacological activity research.

In this study, we summarized 6 types of 191 steroidal saponins of SF-OR based on the identified components and research reports [30,31,32,33]. Most of them were dominated by Type-I spirostanol matric structures, which generally had only one sugar chain. The representative compounds Ophiopogonin A, B, C, and D have a wide range of pharmacological effects, such as hypoglycemia [34], antitumor [35], the protection of myocardial ischemia [36], regulation of immunity [37], and resistance to myocardial infarction [38]. It was found that the steroidal saponin constituents are susceptible to the partial removal of sugar groups during SF and the further formation of corresponding sulfur-containing derivatives. A total of 95 sulfur-containing derivatives of steroidal saponin were found in the identification of SF-OR. It has been shown that SF decreases the total content of polysaccharides and increases the content of oligosaccharides and free monosaccharides in TCM. Although SO_2_ residues decreased during storage, chemical transformations of non-saccharide and sugar components continued to occur in the TCM [39]. In addition, SF induces chemical transformations of the bioactive components in TCM. A reduction in the bioactive components may affect their efficacy, and the generation of new sulfur-containing derivatives may also affect their safety [40]. For example, three highly toxic and carcinogenic components to humans were found in the volatile oil of sulfur-fumigated Radix Angelicae Dahuricae [41]. The anti-inflammatory and anti-tumor effects of sulfur-fumigated Astragalus membranaceus were substantially reduced [42]. The analgesic effect of Paeoniae Radix Alba was reduced after SF [43]. Therefore, when applying sulfur-fumigated TCM, attention should be paid to the transformation of its chemical composition and safety evaluation.

So far, there is very limited information on SF-induced changes in the steroidal saponin composition of OR, which can be analyzed quickly and efficiently using our proposed strategy. It provides a model for the identification and pharmacological activity studies of other sulfur-fumigated TCM in the future.

## 4. Materials and Methods

### 4.1. Chemicals and Reagents

The NSF-OR sample was purchased from Beijing Tong Ren Tang in China. The voucher specimen of NSF-OR was deposited in the School of Chinese Pharmacy, Beijing University of Chinese Medicine, Beijing, China.

Methanol and acetonitrile were MS-grade and purchased from Fisher Scientific (Fair Lawn, NJ, USA). Formic acid (MS grade) was provided by Sigma Aldrich (St. Louis, MO, USA). All the other analytical-grade chemicals were available at the workstation of Beijing Chemical Works (Beijing, China). The deionised water used throughout the experiment was purified using the Milli-Q Gradient A 10 System (Millipore, Billerica, MA, USA). Grace Pure SPE C_18_-Low solid-phase extraction cartridges (200 mg/3 mL, 59 μm, 70 Å) were purchased from Grace Davison Discovery Science (Deerfield, IL, USA).

Reference information is provided below. Ophiogenin 3-*O*-α-l-rhamnopyranosyl-(1→2)-β-d-glucopyranoside (CAS:128502-94-3; purity ≥ 98%); 14-hydroxy Sprengerinin C (CAS:1111088-89-1; ≥98%); Pennogenin 3-*O*-[α-l-rhamnopyranosyl-(1→2)][β-d-xylopyranosyl-(1→4)]-β-d-glucopyranoside (CAS:1029017-75-1; ≥98%); Sprengerinin C (CAS:88861-91-0; ≥98%); Ophiopogonin D(CAS:41753-55-3; ≥98%); Ophiopogonin D’(CAS:65604-80-0; ≥98%); Ophiopojaponin C(CAS:911819-08-4; ≥98%).

12-hydroxy Ophiogenin 3-*O*-α-l-rhamnopyranosyl-(1→2)-β-d-glucopyranoside, 14-hydroxydiosgenin-3-*O*-α-l-rhamnopyranosyl-(1→2)-β-d-glucopyranoside, and Pennogenin 3-*O*-[2-*O*-acetyl-α-l-rhamnopyranosyl-(1→2)][β-d-xylopyranosyl-(1→4)]-β-d-glucopyranoside were isolated and identified by the team during the preliminary work. Their structures were fully elucidated by comparing their spectral data (ESI-MS and ^1^H, ^13^C-NMR spectroscopy) with the literature [44].

### 4.2. Preparation of SF-OR Using Laboratory Simulation Method

NSF-OR (100 g) was wetted with 100 mL of water and then left to stand for 0.5 h. Sulfur powders (10 g) were heated until they burned, and then the burning sulfur and wetted OR samples were, respectively, put into the lower and upper layer of a desiccator. The desiccator was kept closed for 24 h. The SF-OR samples were dried in a ventilated drying oven at 50 °C for 24 h [5,45].

### 4.3. Preparation of SF-OR Extract Samples

The SF-OR samples were pulverised into powder (80 mesh). The SF-OR powder was accurately weighed (3 g) and then ultrasonically extracted (250 W, 40 kHz) with 30 mL of 70% methanol for 30 min at room temperature. The supernatant of the extracts was filtered and concentrated into a volume of 3 mL.

### 4.4. Solution Preparation

#### 4.4.1. Sample Solution

The SF-OR extract samples (1 mL) were added to solid-phase extraction (SPE) cartridges pretreated with methanol (5 mL) and deionised water (5 mL), respectively. Then, the SPE cartridges were successively washed with deionised water (3 mL) and methanol (2 mL). The methanol eluent was collected and filtered by a 0.22 μm syringe filter before the UHPLC-LTQ-Orbitrap-MS/MS analysis.

#### 4.4.2. Standard Solution

Each reference standard was accurately weighed and dissolved in methanol to produce the standard solutions, which were stored in the refrigerator at 4 °C before analysis. The mixed standard solution was diluted into a suitable working solution before using it.

### 4.5. Instruments and Analytical Conditions

Chromatographic separation was performed on a Thermo Fisher DIONEX Ultimate 3000 UHPLC system (Thermo Fisher Company, Waltham, MA, USA). An ACQUITY UPLC HSS T3 column (2.1 mm × 100 mm, 1.8 µm) was used. The separation flow rate was 0.3 mL/min and the column temperature was maintained at 25 °C. The mobile phase was water containing 0.1% formic acid (A) and acetonitrile containing 25% methanol (B). The gradient elution conditions were set as follows: 0–2 min, 0–10% B; 2–10 min, 10–20% B; 10–28 min, 20–26% B; 28–31 min, 26% B; 31–45 min, 26–32% B; 45-55 min, 32–46% B; 55–90 min, 46–67% B; 90–93 min, 67–80% B. Mass spectrometry analyses were performed on an LTQ XL™ linear ion trap mass spectrometer (Thermo Fisher Company, Waltham, MA, USA) using an electrospray ionisation (ESI) source. The ion source parameters are listed as follows: the sheath gas and auxiliary gas at a flow rate of 30 and 10 (arbitrary units), respectively, capillary temperature of 350 °C, capillary voltage of −35 V, spray voltage of −3000 V, and collision energy of 35%. HRMS were obtained with full scans at a 30,000 resolution in the mass range of *m*/*z* 100–800.

### 4.6. Peak Selections and Data Processing

A Thermo Xcalibur 2.1 workstation was used for the data acquisition and processing. Peaks with an intensity over 10,000 in negative ion mode were selected as fragment ions. The chemical formulas attributed to the selected peaks were calculated using a formula predictor by setting the parameters as follows: C [5–60], H [5–100], O [1–40], S [0–2], and the ring double-bond (RDB) equivalent value [3–30]. The maximum mass errors between the measured and calculated values were fixed within 5 ppm. All the relevant data, including the peak number, retention time, accurate mass, predicted chemical formula, and corresponding mass error, were recorded.

## 5. Conclusions

In this study, an analytical strategy for PIL-DE acquisition combined with DPIs and NLFs based on UHPLC-LTQ-Orbitrap MS was proposed. Steroidal saponins and their transformed components in SF-OR were analyzed according to the method. As a result, 191 steroidal saponins were quickly screened and identified from SF-OR. There were 105 Type-I, 12 Type-II and Type-III, 13 Type-IV, 18 Type-V, and 43 Type-VI. In addition, the occurrence of sulfate or sulfite esterification reactions led to the emergence of many new sulfur-containing derivatives in the SF process. Based on the summarized MS cleavage patterns and specific isotope peaks of the steroidal saponins, 95 sulfated and sulfite-esterified components of steroidal saponins were identified from the SF-OR, including 29 Type-I, 3 Type-II and Type-III, 8 Type-V, and 55 Type-VI. The implementation of this new strategy comprehensively characterized the compositional profile of the steroidal saponins in SF-OR. Meanwhile, this case also confirmed the feasibility of the new strategy for the identification of the chemical compositions of SF-herbs, which provides a reference for the compositional identification of other Chinese herbal medicines.

## Figures and Tables

**Table 1:** NLFs of OR Steroidal Saponins.

Name	NLF (Da)	Formula
Glc	162	C_6_H_10_O_5_
Xyl	132	C_5_H_8_O_4_
Ara	132	C_5_H_8_O_4_
Rha	146	C_6_H_10_O_4_
Fuc	146	C_6_H_10_O_4_
Ac	42	C_2_H_2_O
AcO	46	CH_2_O_2_
Glc + Rha	308	C_12_H_20_O_9_
Glc + Glc	324	C_12_H_20_O_10_
Rha + Fuc	192	C_12_H_20_O_8_
Rha + Xyl	278	C_11_H_18_O_8_
Rha + Xyl + Ara	310	C_16_H_26_O_12_
Glc + Rha + Xyl	440	C_17_H_28_O_13_
Rha + Fuc + Xyl	456	C_17_H_28_O_12_
Glc + Glc + Rha	470	C_18_H_30_O_14_
Glc + Glc + Glc + Rha	632	C_24_H_40_O_19_
Rha + Fuc + Xyl + Ara	588	C_22_H_36_O_19_
Glc + Glc + Glc + Xyl	618	C_23_H_38_O_19_
Glc + Glc + Xyl + Rha	602	C_23_H_38_O_18_
Glc + Glc + Glc + Xyl + Rha	764	C_29_H_48_O_24_

**Table 2:** *p*DPIs of OR Steroidal Saponins.

Type	*p*DPIs	The Core Structure
I	707(C_38_H_59_O_12_)/575(C_33_H_51_O_18_)	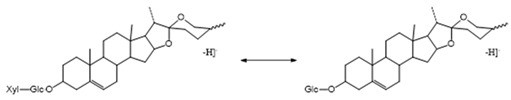
	721(C_39_H_61_O_12_)/575(C_33_H_51_O_8_)	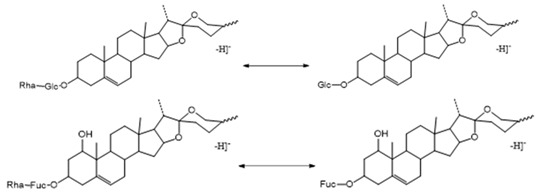
	723(C_38_H_59_O_13_)/591(C_33_H_51_O_9_)	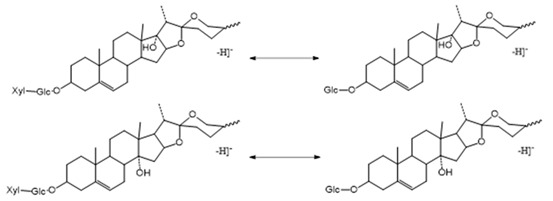
	737(C_39_H_61_O_13_)/591(C_33_H_51_O_9_)	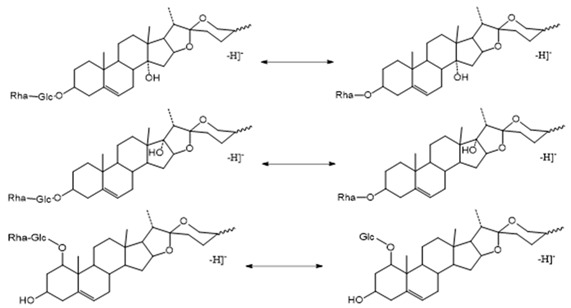
	739(C_38_H_59_O_14_)/607(C_33_H_51_O_10_)	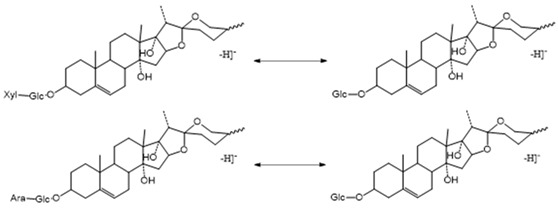
	753(C_39_H_61_O_14_)/607(C_33_H_51_O_10_)	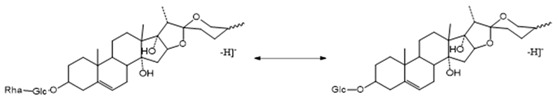
	755(C_38_H_59_O_15_)/623(C_33_H_51_O_11_)	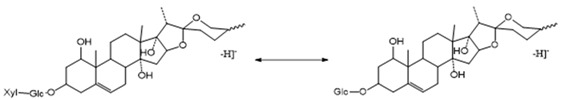
	707(C_38_H_59_O_12_)/561(C_32_H_49_O_8_)	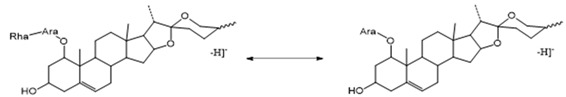
	769(C_39_H_61_O_15_)/623(C_33_H_51_O_11_)	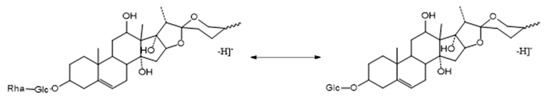
II&III	719(C_39_H_59_O_12_)/ 573(C_33_H_51_O_8_)	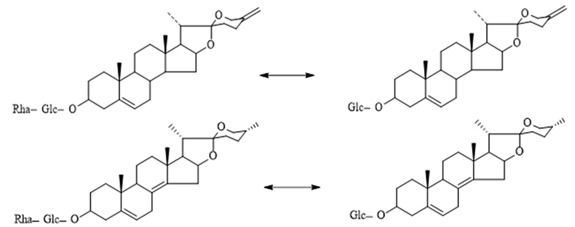
	735(C_39_H_59_O_13_)/ 589(C_33_H_51_O_9_)	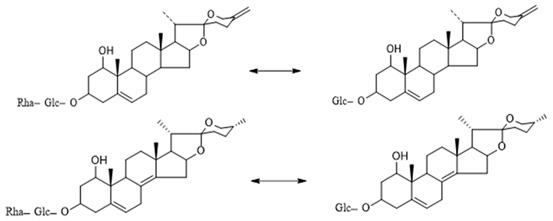
IV	769(C_39_H_61_O_15_)/ 589(C_33_H_49_O_9_)	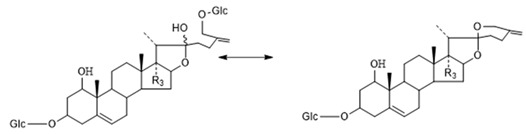
V	753(C_39_H_61_O_14_)/ 573(C_33_H_49_O_8_)	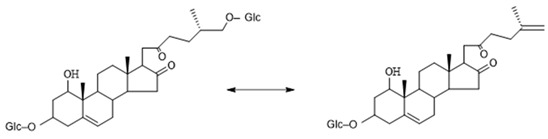
VI	755(C_39_H_63_O_14_)/ 575(C_33_H_51_O_8_)	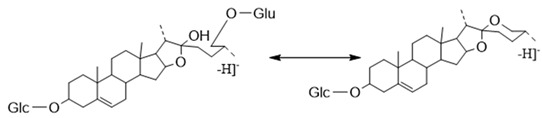
	771(C_39_H_63_O_15_)/591(C_33_H_51_O_9_)	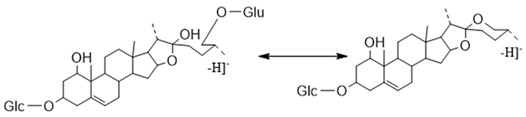
	787(C_39_H_63_O_16_)/ 607(C_33_H_51_O_10_)	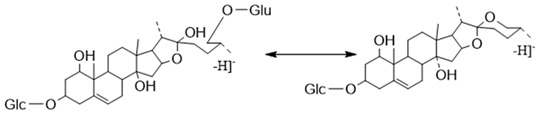

**Table 3:** Identification results of prototype components of Steroid Saponins in SF-OR.

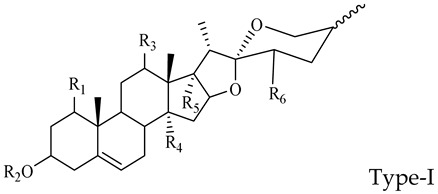										
NO.	Mass (*m*/*z*)	Formula [M − H]^−^	MS/MS Fragment Ions	Identifification	R_1_	R_2_	R_3_	R_4_	R_5_	R_6_
S1	707.4011	C_38_H_59_O_12_	MS^2^[707]: 575(100)	Sprengerinin A		Xyl-glc				
S2–S8	721.4157	C_39_H_61_O_12_	MS^2^[721]: 575(100)	Ophiopogonin C’		Rha-glc				
	721.4157	C_39_H_61_O_12_	MS^2^[721]: 575(100)	Ophiopogonin B	*O*-Rha-fuc					
	721.4157	C_39_H_61_O_12_	MS^2^[721]: 575(100)	Nolinpspiroside F	*O*-Fuc	Rha				
S9	723.3950	C_38_H_59_O_13_	MS^2^[723]: 591(100)	Ophiopogonin E		Xyl-glc			OH	
S10	723.3950	C_38_H_59_O_13_	MS^2^[723]: 591(100)	Ophiopogonin S		Xyl-glc		OH		
S11–S15	737.4106	C_39_H_61_O_13_	MS^2^[737]: 247(100), 591(74), 693(14), 367(8), 424(7), 265(6)	14-Hydroxydiosgenin 3-*O*-α-l-Rha-(1→2)-β-d-Glc		Rha-glc		OH		
	737.4106	C_39_H_61_O_13_	MS^2^[723]: 591(100)	Pennogenin 3-*O*-α-l-Rha-(1→2)-β-d-Glc		Rha-glc			OH	
	737.4106	C_39_H_61_O_13_	MS^2^[723]: 591(100)	Floribundasaponin B	*O*-Rha-glc				OH	
S16	739.3899	C_38_H_59_O_14_	MS^2^[739]: 607(100)	Ophiogenin 3-*O*-xyl(1→4) β-d-glc		Xyl-glc		OH	OH	
	739.3899	C_38_H_59_O_14_	MS^2^[739]: 607(100)	Bornyl 7-*O*-α-l-Ara(1→6)-β-d-Glc		Ara-glc		OH	OH	
S17–S18	749.4101	C_40_H_61_O_13_	MS^2^[749]: 707(100), 689(44), 575(30)	Ac-Sprengerinin A		Ac-Xyl(1→4)glc				
S19–S23	753.4055	C_39_H_61_O_14_	MS^2^[753]: 607(100), 445(20)	14-Hydroxydiosgenin 3-*O*-α-l-Glc-(1→2)-β-d-Glc		Glc-glc		OH		
	753.4055	C_39_H_61_O_14_	MS^2^[753]: 607(100), 445(20)	Pennogenin 3-*O*-α-l-Glc-(1→2)-β-d-glc		Glc-glc			OH	
	753.4055	C_39_H_61_O_14_	MS^2^[753]: 607(100), 445(20)	Ophiogenin 3-*O*-α-l-Rha(1→2)-β-d Glc		Rha-glc		OH	OH	
S24	755.3848	C_38_H_59_O_15_	MS^2^[755]: 623(100)	—		Xyl-glc		OH		OH
S25–S26	763.4257	C_41_H_63_O_13_	MS^2^[763]: 721(100), 703(16), 575(10)	Ophiopogonin A	*O*-Ac-rha-fuc					
S27	765.4050	C_40_H_61_O_14_	MS^2^[765]: 723(100), 705(39), 719(22), 591(20)	Ac-Ophiopogonin E		Ac-Xyl-glc			OH	
S28	769.4004	C_39_H_61_O_15_	MS^2^[769]: 623(100), 443(9), 605(5), 461(5)	—		Rha-glc	OH	OH	OH	
S29–S34	779.4206	C_41_H_63_O_14_	MS^2^[779]: 737(100), 719(17), 591(10)	Ac-(S11–S15)		Rha-glc		OH		
S35–S38	795.4155	C_41_H_63_O_15_	MS^2^[795]: 753(100), 735(36), 607(30), 445(10)	Ac-Ophiogenin 3-*O*-α-l-Rha(1→2)-β-d-Glc		Ac-Rha-glc		OH	OH	
	795.4155	C_41_H_63_O_15_	MS^2^[795]: 753(100), 735(36), 591(23)	Ac-pennogenin 3-*O*-α-l-Glc-(1→2)-β-d-glc		Ac-Glc-glc			OH	
S39	839.4423	C_43_H_67_O_16_	MS^2^[839]: 707(100), 561(6)	Ruscogenin 1-*O*-α-l-Xyl(1→4)Rha(1→2)Ara	*O*-xyl-rha-ara					
S40–S43	853.4580	C_44_H_69_O_16_	MS^2^[853]: 721(100), 707(10), 575(6)	LS-10	*O*-rha-xyl-fuc					
	853.4580	C_44_H_69_O_16_	MS^2^[853]: 721(100), 575(6)	Ophiopogonin D	*O*-rha-xyl-fuc					
	853.4580	C_44_H_69_O_16_	MS^2^[853]: 721(100), 575(10)	Ophiopogonin D′		Rha-xyl-glc				
	853.4580	C_44_H_69_O_16_	MS^2^[853]: 721(100), 575(8)	Sprengerinin C		Rha-xyl-glc				
S44–S53	869.4529	C_44_H_69_O_17_	MS^2^[869]: 737(100), 738(45), 591(2)	(25*R*)-Ruscogenin 3-yl α-l-Rha-(1→2)-[β-d-Xyl-(1→4)]-β-d-Glc	OH	Rha-xyl-glc				
	869.4529	C_44_H_69_O_17_	MS^2^[869]: 737(100), 591(2)	Pennogenin 3-*O*-α-l-Rha-(1→2)-[β-d-Xyl-(1→4)]-β-d-Glc		Rha-xyl-glc			OH	
	869.4529	C_44_H_69_O_17_	MS^2^[869]: 737(100), 738(45), 591(2)	14-Hydroxy Sprengerinin		Rha-xyl-glc		OH		
S54	883.4685	C_45_H_71_O_17_	MS^2^[883]: 737(100), 571(34), 557(10)	Rha-(S11–S15)		Rha-glc-glc				
S55–S58	885.4478	C_44_H_69_O_18_	MS^2^[885]: 753(100), 607(2)	Cixi-ophiopogon A		Rha-xyl-glc		OH	OH	
	885.4478	C_44_H_69_O_18_	MS^2^[885]: 753(100), 607(4), 735(3), 445(2)	Ophiopojaponin C		Rha-xyl-glc		OH	OH	
S59–S69	895.4685	C_46_H_71_O_17_	MS^2^[895]: 853(100), 835(94), 721(15), 707(3)	Ophiopogonin C	*O*-Rha-xyl-glc	Ac				
	895.4685	C_46_H_71_O_17_	MS^2^[895]: 853(100), 835(59), 721(16), 763(5), 707(3)	Ophiopogonin B′		Ac-rha-xyl-glc				
	895.4685	C_46_H_71_O_17_	MS^2^[895]: 853(100), 835(59), 721(16), 763(4), 707(4)	Diosgenin 3-*O*-[2-*O*-Ac-α-l-Rha-(1→2)][β-d-Xyl-(1→4)]-β-d-Glc		Ac-rha-xyl-glc				
	895.4685	C_46_H_71_O_17_	MS^2^[895]: 853(100), 835(52), 721(15), 707(4), 763(3)	Ophiopogonin P		Ac-rha-xyl-glc				
	895.4685	C_46_H_71_O_17_	MS^2^[895]: 853(100), 835(47), 721(15), 707(5), 763(3)	Ophiopogonin Q		Ac-rha-xyl-glc				
S68	901.4427	C_44_H_69_O_19_	MS^2^[901]: 769(100), 623(30), 751(10)	Xyl-S28		Rha-xyl-glc	OH	OH	OH	
S69–S80	911.4634	C_46_H_71_O_18_	MS^2^[911]: 869(100), 851(49), 737(11), 723(3), 591(2)	Pennogenin 3-*O*-α-l-Rha-(1→2)-β-d-Xyl-(1→4)-β-d-Glc	Ac-Rha-xyl-glc			OH		
	911.4634	C_46_H_71_O_18_	MS^2^[911]: 869(100), 851(58), 737(21), 779(10), 723(8), 652(6), 719(6)	Ac-Ruscogenin 3-*O*-α-l-Rha-(1→2)-β-d-Xyl-(1→4)-β-d-Glc	[Ac-rha(1→2)]xyl(1→4)glc	OH				
	911.4634	C_46_H_71_O_18_	MS^2^[911]: 869(100), 851(63), 737(15), 779(6)	Ophiopojaponin A		[Ac-rha(1→2)]xyl(1→4)glc			OH	
S81–S84	915.4584	C_45_H_71_O_18_	MS^2^[915]: 769(100), 753(56), 589(13), 735(11), 607(6)	(25*R*)-14α,17α-Hydroxyspirost-5-en-3β-yl3-*O*-α-l-Rha-(1→2)-β-d-Glc-(1→3)-β-d-Glc		Rha(1→2)glc(1→3)glc		OH	OH	
S85	917.4740	C_45_H_73_O_19_	MS^2^[917]: 707(100), 871(81), 465(12), 561(10)	Ruscogenin 1-*O*-α-l-Xyl(1→3)Rha(1→2)Ara	*O*-Xyl(1→3)rha(1→2)ara	OH?	OH			
S86–S90	927.4584	C_46_H_71_O_19_	MS^2^[927]: 885(100), 867(54), 753(16), 885(13), 735(7), 739(5), 721(3), 607(2)	Ac-(S55–S58)		Ac-Rha-xyl-glc		OH	OH	
S91	931.4533	C_45_H_71_O_20_	MS^2^[931]: 785(100), 769(56), 623(45), 461(4)	Glc-S28	Rha-glc-glc		OH	OH	OH	
S92–S100	953.4740	C_48_H_73_O_19_	MS^2^[953]: 911(100), 893(64), 851(59), 869(12), 833(9), 737(7)	Ac-(S69–S80)	2Ac-Rha-xyl-glc?			OH		
S101–S102	969.4689	C_48_H_73_O_20_	MS^2^[969]: 927(100), 909(46), 867(38), 849(18), 885(13), 753(10)	Ac-(S86–S90)		2Ac-Rha-xyl-glc		OH	OH	
S103	997.5002	C_50_H_77_O_20_	MS^2^[997]: 853(100), 895(7), 835(5), 721(3)	Xyl-(S40–S43)	*O*-rha-xyl-fuc?					
S104–S105	1045.5208	C_51_H_81_O_22_	MS^2^[1045]: 833(100), 899(33), 737(22), 719(8)	—	Glc-rha	Glc-glc	OH			
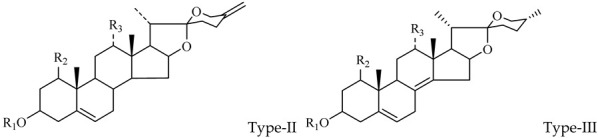										
**NO.**	**Mass (*m*/*z*)**	**Formula [M − H]^−^**	**MS/MS Fragment Ions**	**Identifification**	**R_1_**		**R_2_**		**R_3_**	
S106	719.4001	C_39_H_59_O_12_	MS^2^[719]: 573(100)	(1β,3β)-3-Hydroxyspirosta-5,25(27)-dien-1-yl *O*-6-deoxy-α-l-Rha-(1→2)-β-d-Glc	Rha(1→2)glc					
S107–S108	807.4161	C_42_H_63_O_15_	MS^2^[807]: 719(100), 761(77), 683(13)	Ac-(1β,3β)-3-Hydroxyspirosta-5,25(27)-dien-1-yl *O*-6-deoxy-α-l-Rha-(1→2)-β-d-Glc+ HCOOH	Ac-Rha(1→2)glc +HCOOH					
S109–S112	851.4418	C_44_H_67_O_16_	MS^2^[851]: 719(100), 573(45)	(1β,3β)-3-Hydroxyspirosta-5,25(27)-dien-1-yl *O*-6-deoxy-α-l-Rha-(1→2)-*O*-[β-d-Xyl-(1→4)]-β-d-Fuc/25(R)-spirost-5,8-diene-3β-ol-3-*O*-α-l-Rha(1→2)-β-d-Xyl(1→4)-β-d-Glc	Xyl(1→3)rha(1→2)glc					
S113–S114	867.4372	C_44_H_67_O_17_	MS^2^[867]: 735(100), 721(3), 523(2), 589(2)	(1β,3β)-3-Hydroxyspirosta-5,25(27)-dien-1-yl *O*-6-deoxy-α-l-Rha-(1→2)-*O*-[β-d-Xyl-(1→4)]-β-d-Glc	xyl(1→4)rha(1→2)glc		OH?			
S115	881.4529	C_45_H_69_O_17_	MS^2^[881]: 735(100)	(1β,3β)-3-Hydroxyspirosta-5,25(27)-dien-1-yl *O*-6-deoxy-α-l-Rha-(1→2)-*O*-[β-d-Fuc-(1→4)]-β-d-Glc	Rha-glc-fuc		OH?			
S116–S117	1191.5429	C_56_H_87_O_27_	MS^2^[1191]: 1059(100), 1041(61), 1029(61), 1045(48), 913(40), 897(34), 879(30), 895(23), 733(20), 571(20)	—	Xyl-rha-glc		Glc-glc			
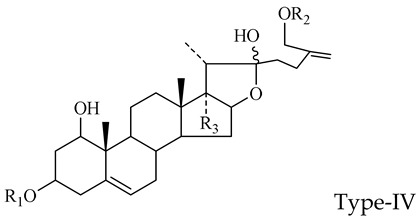										
**NO.**	**Mass (*m*/*z*)**	**Formula [M − H]^−^**	**MS/MS Fragment Ions**	**Identifification**	**R_1_**		**R_2_**		**R_3_**	
S118	915.4584	C_45_H_71_O_19_	MS^2^[915]: 769(100), 751(77), 589(28), 897(20)	26-*O*-β-d-Glc-20α-hydroxyfurost-25,27-dine-3-α-l-Rha-β-d-Glc	Rha-glc		Glc			
S119–S120	915.4584	C_45_H_71_O_19_	MS^2^[915]: 753(100), 733(90), 879(45), 573(30)	26-*O*-β-d-Glc-β-d-Glc-20α-hydroxyfurost-25,27-dine-3-α-l-Rha	Rha		Glc-glc			
S121–S124	1047.5007	C_50_H_79_O_23_	MS^2^[1047]: 915(100), 901(19), 769(16), 897(15), 589(5)	Xyl-S118	Rha-xyl-glc		Glc			
	1047.5007	C_50_H_79_O_23_	MS_2_[1047]: 915(100), 769(14)	Ara-S118	Rha-Ara-glc		Glc			
S125–S126	1077.5476	C_52_H_85_O_23_	MS_2_[1077]: 915(100), 931(25), 769(20), 897(10), 589(7)	Glc-(S119-S120)	Rha-glc		Glc-glc			
S127	1195.5378	C_55_H_87_O_28_	MS_2_[1195]: 1063(100), 917(27), 901(20)	Xyl-Ara-(S119-S120)	Xyl-rha-ara		Glc-glc		OH	
S128–S130	1209.5535	C_56_H_89_O_28_	MS^2^[1209]: 1077(100), 915(84), 1047(67), 1063(66), 901(22), 915(19), 769(15), 751(14)	Xyl-Glc-(S119-S120)	Rha-xyl-glc		Glc-glc			
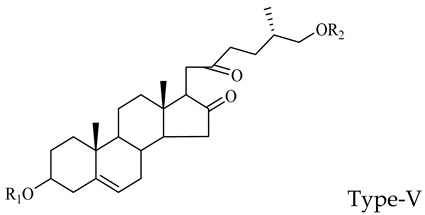										
**NO.**	**Mass (*m*/*z*)**	**Formula [M − H]^−^**	**MS/MS Fragment Ions**	**Identifification**	**R_1_**				**R_2_**	
S131	899.4634	C_45_H_71_O_18_	MS^2^[899]: 753(100), 573(26), 735(7), 737(6), 591(5)	(20*R*,25*R*)-26-*O*-β-d-Glc-3β,26-dihydroxycholest-5-en-16,22-dioxo-3-*O*-α-l-Rha(1→2)-β-d-Glc	Rha-glc				Glc	
S132–S134	1031.5057	C_50_H_79_O_22_	MS^2^[1031]: 899(100), 885(14), 881(11), 753(11)	(20*R*,25*R*)-26-*O*-β-d-glc-3β,26-dihydroxycholest-5-en-16,22-dioxo-3-*O*-α-l-Rha(1→2)-[β-d-Xyl(1→3)]β-d-Glc	Rha-xyl-glc				Glc	
S135–S142	1061.5158	C_51_H_81_O_23_	MS^2^[1061]: 899(100), 915(30), 753(25), 735(15), 573(10), 591(4)	Glc-(20*R*,25*R*)-26-*O*-β-d-glc-3β,26-dihydroxycholest-5-en-16,22-dioxo-3-*O*-α-l-Rha(1→2)-β-d-Glc	Rha-glc				Glc-glc	
S143–S148	1193.5586	C_56_H_89_O_27_	MS^2^[1193]: 1061(100), 899(85), 1047(63), 1031(49), 915(21), 753(19), 885(17), 881(13)	Xyl-Glc-(20*R*,25*R*)-26-*O*-β-d-Glc-3β,26-dihydroxycholest-5-en-16,22-dioxo-3-*O*-α-LRha(1→2)-β-d-Glc	Rha-xyl-glc				Glc-glc	
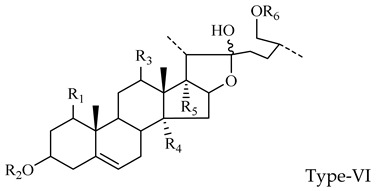										
**NO.**	**Mass (*m*/*z*)**	**Formula [M − H]^−^**	**MS/MS Fragment Ions**	**Identifification**	**R_1_**	**R_2_**	**R_3_**	**R_4_**	**R_5_**	**R_6_**
S149–S150	901.4791	C_45_H_73_O_18_	MS^2^[901]: 755(100), 593(4), 575(3), 737(3)	—		Rha-glc				glc
S151–S153	917.4740	C_45_H_73_O_19_	MS^2^[917]: 771(100), 591(13), 755(3), 429(2)	Ophiofurspiside M	OH	Rha-glc				Glc
	917.4740	C_45_H_73_O_19_	MS^2^[917]: 771(100), 591(22), 755(3)	Ophiopojaponin B		Rha-glc		OH		Glc
S154–S156	933.4689	CH_7345_O_20_	MS^2^[933]: 787(100), 607(34), 445(14)	Ophiofurspiside F/isomer		Rha-glc		OH	OH	Glc
S157–S158	943.4897	C_47_H_75_O_19_	MS^2^[943]: 883(100), 901(65), 775(16)	—		Ac-Rha-glc				glc
S159–S162	959.4846	C_47_H_75_O_20_	MS^2^[959]: 899(100), 917(42)	Ac-Ophiofurspiside M/Ophiopojaponin B	OH?	Rha-glc				Glc
S163–S164	1033.5213	C_50_H_81_O_22_	MS^2^[1033]: 901(100), 887(42), 883(13), 755(10), 575(5)	Ophiopogonin T		Rha-xyl-fuc				Glc
	1033.5213	C_50_H_81_O_22_	MS^2^[1033]: 901(100), 755(50), 887(42), 883(13)	Ophiopogoside A		Ara-rha-glc				Glc
S165–S166	1049.5163	C_50_H_81_O_23_	MS^2^[1049]: 917(100), 771(55), 903(51), 899(16), 754(5), 591(3)	Xyl-Ophiofurspiside M	OH	Xyl-rha-glc				Glc
	1049.5163	C_50_H_81_O_23_	MS^2^[1049]: 917(100), 771(60), 903(51), 899(16), 754(5), 591(3)	Xyl-Ophiopojaponin B		Xyl-rha-glc		OH		Glc
S167	1063.5304	C_51_H_83_O_23_	MS^2^[1063]: 901(100), 737(67), 755(56), 623(23)	Trigoneoside Iva/isomer		Rha-glc				Glc-glc
S168	1065.5112	C_50_H_81_O_24_	MS^2^[1065]: 933(100), 919(47), 787(45), 771(9), 915(9), 607(5)	Xyl-Ophiofurspiside F		Xyl-rha-glc		OH	OH	glc
S169	1075.5320	C_52_H_83_O_23_	MS^2^[1075]: 1015(100), 1033(78), 883(20), 901(11)	Ac-Ophiopogonin T/Ophiopogoside A		Ac-Rha-xyl-fuc				Glc
S170–S173	1079.5269	C_51_H_83_O_24_	MS^2^[1079]: 917(100), 933(60), 771(59), 591(16), 753(12), 899(3), 755(2)	Glc-Ophiofurspiside M	OH	Rha-glc				Glc-glc
	1079.5269	C_51_H_83_O_24_	MS^2^[1079]: 917(100), 933(60), 771(59), 591(30)	Glc-Ophiopojaponin B		Rha-glc		OH		Glc-glc
S174–S183	1095.5218	C_51_H_83_O_25_	MS^2^[1095]: 933(100), 949(60), 787(59), 769(16), 607(12), 771(8)	Ophiopogonin K		Rha-glc		OH	OH	Glc-glc
	1095.5218	C_51_H_83_O_25_	MS^2^[1095]: 933(100), 949(60), 787(59), 769(16), 607(12), 771(8)	Ophiopogonin K-isomer	OH	Rha-glc		OH		Glc-glc
S184	1195.5378	C_56_H_91_O_27_	MS^2^[1195]: 1063(100), 901(91), 1033(89), 1049(61), 755(50), 887(43), 575(20)	Ophiopogonin F/isomer		Rha-xyl-glc				Glc-glc
S185	1211.5691	C_56_H_91_O_28_	MS^2^[1211]: 1079(100), 1049(70), 917(66), 1065(57), 771(37), 903(32), 933(31), 753(15), 1061(13), 899(12), 591(8)	Ophiopogonin J		Rha-xyl-glc		OH		Glc(1-2)glc
S186–S188	1211.5691	C_56_H_91_O_28_	MS^2^[1211]: 1079(100), 1049(57), 917(50), 1065(47), 771(30), 903(32), 933(31), 753(15), 1061(13), 899(12), 591(8)	Ophiopogonin N		Rha-xyl-glc		OH		Glc(1-6)glc
	1211.5691	C_56_H_91_O_28_	MS^2^[1211]: 1079(100), 1049(68), 917(60), 771(45), 903(32), 933(31), 899(12), 591(10), 429(5)	Ophiorospiside C		Rha-xyl-glc			OH	Glc-glc
S189–S190	1227.5641	C_56_H_91_O_29_	MS^2^[1227]: 1095(100), 1065(87), 933(71), 1081(53), 919(39), 949(26), 787(22), 1077(15), 769(15), 915(13), 607(10)	Hydroxyl-Ophiopogonin J/Ophiopogonin N/Ophiorospiside C		Rha-xyl-glc		OH?	OH?	Glc-glc
S191	1237.5848	C_58_H_93_O_28_	MS^2^[1237]: 1195(100), 1177(98), 1045(11), 1033(9), 1015(7), 1063(5), 883(3), 899(1)	Ac-Ophiopogoin F/isomer		Ac-Rha-Xyl-glc				Glc-glc

Note: “_”: DPI; “?”: Unable to determine the binding position; “-”: Unable to determine the name of the compound.

**Table 4:** Identification results of sulfur-containing derivatives of Steroid Saponins in SF-OR.

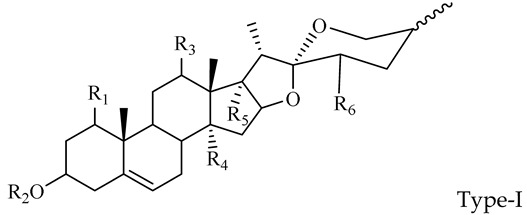											
NO.	Mass (*m*/*z*)	Formula [M − H]^−^	MS/MS Fragment Ions	Identifification	SO_3_/SO_2_	R_1_	R_2_	R_3_	R_4_	R_5_	R_6_
SS1	639.3191	C_33_H_51_O_10_S	MS^2^[639]: 493(100), 475(77), 535(66), 177(55)	Diosgenin 1-*O*-α-l-Fuc/isomer-sulfate	SO_3_	*O*-Fuc					
SS2	655.3141	C_33_H_51_O_11_S	MS^2^[655]: 509(100), 491(13), 551(9)	Ruscogenin 1-*O*-α-l-Fuc/isomer-sulfate	SO_3_	*O*-Fuc	OH				
SS3–SS6	671.3090	C_33_H_51_O_12_S	MS^2^[671]: 509(100), 551(28), 653(11), 510(11), 493(9)	Ruscogenin 3-α-l-Glc/isomer-sulfate	SO_3_	OH	Glc				
SS7	687.3039	C_33_H_51_O_13_S	MS^2^[687]: 525(100)	Ophiogenin 3-*O*-β-d-Glc-sulfate	SO_3_		Glc		OH	OH	
SS8	787.3563	C_38_H_59_O_15_S	MS^2^[787]: 357(100), 641(91), 683(23), 642(11), 385(3), 713(3)	Ruscogenin 3-α-l-Ara-Rha/isomer-sulfate	SO_3_	OH	Rha-ara				
SS9	801.3714	C_39_H_61_O_15_S	MS^2^[801]: 655(100), 801(42), 656(14), 804(11)	Ophiopogonin B/C’/isomer-sulfate	SO_3_	OH	Rha-fuc				
SS10–SS14	817.3663	C_39_H_61_O_16_S	MS^2^[817]: 671(100), 771(69), 336(46)	Pennogenin 3-*O*-α-l-Rha-(1→2)-β-d-Glc/Floribundasaponin B/isomer-sulfate	SO_3_		Rha-glc			OH	
SS15–SS16	833.3618	C_39_H_61_O_17_S	MS^2^[833]: 815(100), 687(73), 387(29), 816(23), 617(13), 673(12), 688(11)	Ophiogenin 3-*O*-α-l-Rha(1→2)-β-D Glc/isomer-sulfate	SO_3_		Rha-glc		OH	OH	
SS17–SS18	933.4137	C_44_H_69_O_19_S	MS^2^[933]: 787(100), 801(5), 509(2)	Ophiopogonin D/isomer-sulfate	SO_3_	*O*-Rha-xyl-fuc					
SS19–SS20	949.4086	C_44_H_69_O_20_S	MS^2^[949]: 803(100), 787(50), 357(47), 845(17), 788(9), 591(8)	(25*R*)-Ruscogenin 3-yl α- L-Rha-(1→2)-[β-d-Xyl-(1→4)]-β-d-Glc/isomer-sulfate	SO_3_	OH	Rha-glc-ara				
SS21–SS27	963.4248	C_45_H_71_O_20_S	MS^2^[963]: 817(100), 801(76), 637(20), 655(17), 843(14)	Pennogenin 3-*O*-α-l-Rha-(1→2)-β-d-Glc-fuc	SO_3_		Fuc-Rha-glc			OH	
SS28	965.4035	C_44_H_69_O_21_S	MS^2^[965]: 819(100), 357(67)	Cixi-ophiopogon A/Ophiopojaponin C/isomer-sulfate	SO_3_		Rha-glc-ara		OH	OH	
SS29	995.4146	C_45_H_71_O_22_S	MS^2^[995]: 849(100), 669(94), 833(87), 687(24), 850(20), 670(18), 834(17), 875(12)	(25*R*)-14α,17α-Hydroxyspirost-5-en-3β-yl3-*O*-α-l-Rha-(1→2)-β-d-Glc-(1→3)-β-d-Glc/isomer-sulfate	SO_3_		Rha(1→2)glc(1→3)glc		OH	OH	
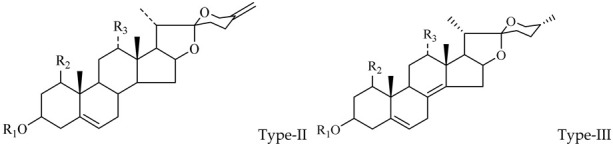											
**NO.**	**Mass (*m*/*z*)**	**Formula [M − H]^−^**	**MS/MS Fragment Ions**	**Identifification**	**SO_3_/SO_2_**	**R_1_**		**R_2_**		**R_3_**	
SS30	799.3563	C_39_H_59_O_15_S	MS^2^[799]: 653(100), 695(20), 371(19), 654(18)	(1β,3β)-3-Hydroxyspirosta-5,25(27)-dien-1-yl-*O*-6-deoxy-α-l-Rha-(1→2)-β-d-Glc/isomer-sulfate	SO_3_	Rha(1→2)glc					
SS31	947.3935	C_44_H_67_O_20_S	MS^2^[947]: 801(100), 785(52), 357(41), 915(20), 843(19), 767(17), 827(10)	(1β,3β)-3-Hydroxyspirosta-5,25(27)-dien-1-yl-*O*-6-deoxy-α-l-Rha-(1→2)-*O*-[β-d-Xyl-(1→4)]-β-d-Glc/isomer-sulfate	SO_3_	xyl(1→4)rha(1→2)glc		OH?			
SS32	961.4086	C_45_H_69_O_20_S	MS^2^[961]: 815(100), 799(54), 781(24), 371(14), 816(14)	(1β,3β)-3-Hydroxyspirosta-5,25(27)-dien-1-yl-*O*-6-deoxy-α-l-Rha-(1→2)-*O*-[β-d-Fuc-(1→4)]-β-d-Glc/isomer-sulfate	SO_3_	Xyl-rha-glc		Glc-glc			
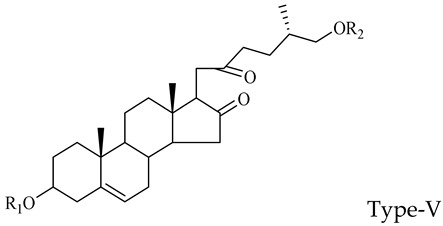											
**NO.**	**Mass (*m*/*z*)**	**Formula [M − H]^−^**	**MS/MS Fragment Ions**	**Identifification**	**SO_3_/SO_2_**	**R_1_**				**R_2_**	
SS33–SS39	963.4248	C_45_H_71_O_20_S	MS^2^[963]: 817(100), 801(76), 637(20), 655(17), 843(14), 818(13)	(20*R*,25*R*)-26-*O*-β-d-Glc-3β,26-dihydroxycholest-5-en-16,22-dioxo-3-*O*-α-l-Rha(1→2)-β-d-Glc/isomer-sulfite	SO_2_	Rha-Glc				Rha	
SS40	979.4192	C_45_H_71_O_21_S	MS^2^[979]: 817(100), 833(76), 653(42), 859(19), 671(13), 818(11), 834(10), 799(5)	(20*R*,25*R*)-26-*O*-β-d-Glc-3β,26-dihydroxycholest-5-en-16,22-dioxo-3-*O*-α-l-Rha(1→2)-β-d-Glc/isomer-sulfate	SO_3_	Rha-Glc				Rha	
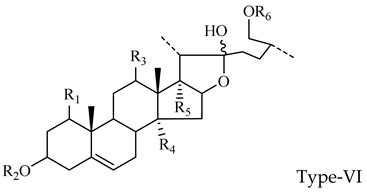											
**NO.**	**Mass (*m*/*z*)**	**Formula [M − H]^−^**	**MS/MS Fragment Ions**	**Identifification**	**SO_3_/SO_2_**	**R_1_**	**R_2_**	**R_3_**	**R_4_**	**R_5_**	**R_6_**
SS41–SS44	819.3825	C_39_H_63_O_16_S	MS^2^[819]: 657(100), 673(43), 447(41), 639(31), 655(24), 699(22), 609(16), 515(14)	—	SO_3_		Glc-fuc				
SS45–SS46	835.3774	C_39_H_63_O_17_S	MS^2^[835]: 673(100), 655(44), 715(20), 674(9)	Hydroxyl-819	SO_3_		Glc-fuc		OH		
SS47	851.3724	C_39_H_63_O_18_S	MS^2^[851]: 623(100), 443(56), 605(20), 769(20)	—	SO_2_	OH	Rha		OH	OH	Glc
SS48–SS51	851.3724	C_39_H_63_O_18_S	MS^2^[851]: 689(100), 671(57), 690(32), 672(20), 731(19)	Dihydroxyl-819	SO_3_		Rha		OH	OH	Glc
SS52–SS53	965.4035	C_45_H_73_O_20_S	MS^2^[965]: 803(100), 785(31), 819(24), 845(15), 755(13), 639(11)	—	SO_2_		Rha-glc				Glc
SS54	967.4197	C_44_H_71_O_21_S	MS^2^[967]: 805(100), 821(79), 689(28), 741(20), 787(19), 447(15)	—	SO_3_	OH	Rha-ara				Glc
SS55–SS60	981.4353	C_45_H_73_O_21_S	MS^2^[981]: 819(100), 801(33), 835(23), 861(17), 895(14), 655(10)	S150-151/isomer-sulfate	SO_3_		Rha-glc				Glc
SS61	997.4303	C_45_H_73_O_22_S	MS^2^[997]: 835(100), 851(36), 817(8), 671(8), 979(7), 591(7), 715(5)	Ophiopojaponin B/isomer-sulfate	SO_3_		Rha-glc		OH		Glc
SS62	1013.4250	C_45_H_73_O_23_S	MS^2^[1013]: 785(100), 851(70), 605(62), 931(32), 623(28), 687(26), 867(23), 443(23), 543(18), 479(18)	Ophiofurspiside F/isomer-sulfate	SO_3_		Rha-glc		OH	OH	Glc
SS63–SS66	1023.4460	C_47_H_75_O_22_S	MS^2^[1023]: 981(100), 893(64), 867(50), 835(40), 863(21), 851(14), 964(11), 903(10)	S158-S159-sulfate	SO_3_		Ac-Rha-glc				Glc
SS67–SS69	1039.4403	C_47_H_75_O_23_S	MS^2^[1039]: 979(100), 909(67), 877(43), 851(38), 879(26), 671(12), 919(9)	Ac-Ophiofurspiside M/Ophiopojaponin B/isomer-sulfate	SO_3_	OH	Rha-glc				Glc
SS70–SS71	1113.4777	C_50_H_81_O_25_S	MS^2^[1113]: 951(100), 967(36), 933(28), 981(24), 655(23), 993(15), 1095(14), 673(10), 771(10)	Ophiopogoside A/Ophiopogonin T/isomer-sulfate	SO_3_		Rha-xyl-fuc				Glc
SS72–SS74	1127.4933	C_51_H_83_O_25_S	MS^2^[1127]: 965(100), 755(9), 803(7), 785(4), 901(4), 981(4)	Trigoneoside Iva/isomer-sulfite	SO_2_		Rha-glc				Glc-glc
SS75–SS76	1129.4726	C_50_H_81_O_26_S	MS^2^[1129]: 967(100), 983(86), 851(30), 1111(28), 997(28), 357(24), 968(19), 984(10)	Xyl-Ophiofurspiside M/isomer-sulfate	SO_3_	OH	Xyl-rha-glc				Glc
SS77–SS80	1143.4882	C_51_H_83_O_26_S	MS^2^[1143]: 981(100), 771(17), 917(8), 819(6), 801(4), 982(4), 997(4),	Glc-Ophiofurspiside M/isomer-sulfate	SO_2_	OH	Rha-glc				Glc-glc
SS81	1145.4675	C_50_H_81_O_27_S	MS^2^[1145]: 785(100), 931(54), 1013(50), 605(39), 983(32), 913(29), 623(29), 587(20), 767(18)	Xyl-Ophiofurspiside F/isomer0sulfate	SO_3_		Xyl-rha-glc		OH	OH	Glc
SS82–SS83	1155.4867	C_52_H_83_O_26_S	MS^2^[1155]: 981(100), 993(74), 1009(39), 835(13), 899(2), 849(2), 655(2)	Ac-Ophiopogoside A/isomer-sulfate	SO_3_		Ara-rha-glc				Glc
SS84–SS87	1159.4831	C_51_H_83_O_27_S	MS^2^[1159]: 997(100), 1162(79), 998(77), 161(55), 1160(31), 999(30), 1013(29), 851(25)	Glc-Ophiofurspiside M/isomer-sulfate	SO_3_	OH	Rha-glc				Glc-glc
SS88–SS89	1175.4785	C_51_H_83_O_28_S	MS^2^[1175]: 1013(100), 785(66), 947(54), 605(41), 767(39), 931(33), 1093(25), 1029(20), 731(17), 569(13), 849(13)	Ophiopogonin K/isomer-sulfate	SO_3_	OH	Rha-glc		OH		Glc-glc
SS90–SS93	1275.5305	C_56_H_91_O_33_S	MS^2^[1275]: 1113(100), 933(80), 1095(71), 735(68), 1129(35), 835(13)	Ophiopogonin F/isomer-sulfate	SO_3_		Rha-xyl-glc				Glc-glc
SS94–SS95	1291.5254	C_56_H_91_O_31_S	MS^2^[1291]: 1129(100), 1145(25), 1013(20), 1130(17), 1159(16), 833(14), 867(10)	Ophiopogonin J/isomer-sulfate	SO_3_		Rha-xyl-glc		OH		Glc-glc

Note: “_”: DPI; “?”: Unable to determine the binding position; “-”: Unable to determine the name of the compound.

## Data Availability

Data are contained within the article and supplementary materials.

## Supplementary Materials

The following supporting information can be downloaded at: https://www.mdpi.com/article/10.3390/molecules29030702/s1, Figure S1: The ESI-MS spectrum (A) and ESI-MS/MS spectrum (B) of ophiopogonin B in negative ion mode; Figure S2: The ESI-MS spectrum (A) and ESI-MS/MS spectrum (B) of Ophiogenin 3-*O*-α-l-rha-(1→2)-β-d-glc in negative ion mode; Figure S3: The ESI-MS spectrum (A) and ESI-MS/MS spectrum (B) of (20*R*,25*R*)-26-*O*-β-d-glc-3β,26-dihydroxycholest-5-en-16,22-dioxo-3-*O*-α-l-rha(1→2)-β-d-Glc in negative ion mode; Figure S4: The ESI-MS spectrum (A) and ESI-MS/MS spectrum (B) of Ophiofurspiside M in negative ion mode; Figure S5: The ESI-MS spectrum (A) and ESI-MS/MS spectrum (B) of Xyl-Ophiofurspiside M in negative ion mode; Figure S6: The ESI-MS spectrum (A) and ESI-MS/MS spectrum (B) of Ophiofurspiside A in negative ion mode; Figure S7: The ESI-MS spectrum (A) and ESI-MS/MS spectrum (B) of Ophiopogonin H in negative ion mode; Figure S8: The ESI-MS spectrum (A) and ESI-MS/MS spectrum (B) of **SS10**–**SS14** in negative ion mode; Figure S9: The ESI-MS spectrum (A) and ESI-MS/MS spectrum (B) of **SS72**–**SS74** in negative ion mode.
