# Supplementary material for: Unveiling the Chemical Composition of Sulfur-Fumigated Herbs: A Triple Synthesis Approach Using UHPLC-LTQ-Orbitrap MS—A Case Study on Steroidal Saponins in Ophiopogonis Radix
Source: Molecules. 2024 Feb 2;29(3):702. doi: 10.3390/molecules29030702 (PMC10856428; doi:10.3390/molecules29030702)
Supplement: Supplementary file 1 [file molecules-29-00702-s001.zip › molecules-2829282-supplementary.pdf]

Supplement

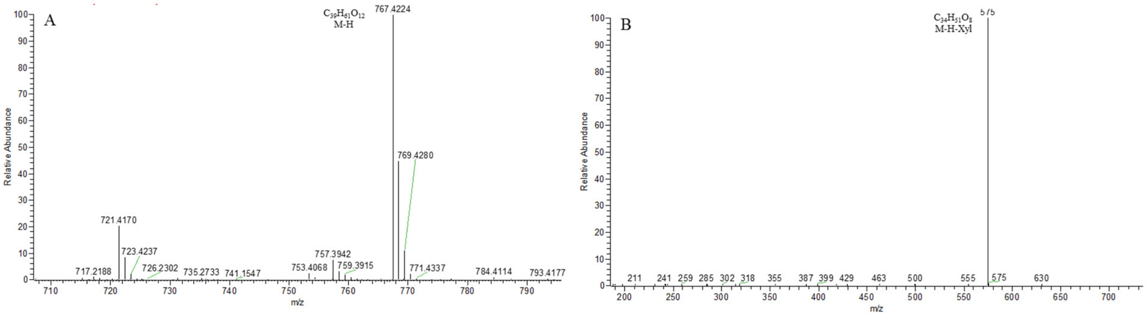

Figure S1. The ESI-MS spectrum (A) and ESI-MS/MS spectrum (B) of ophiopogonin B in negative ion mode.

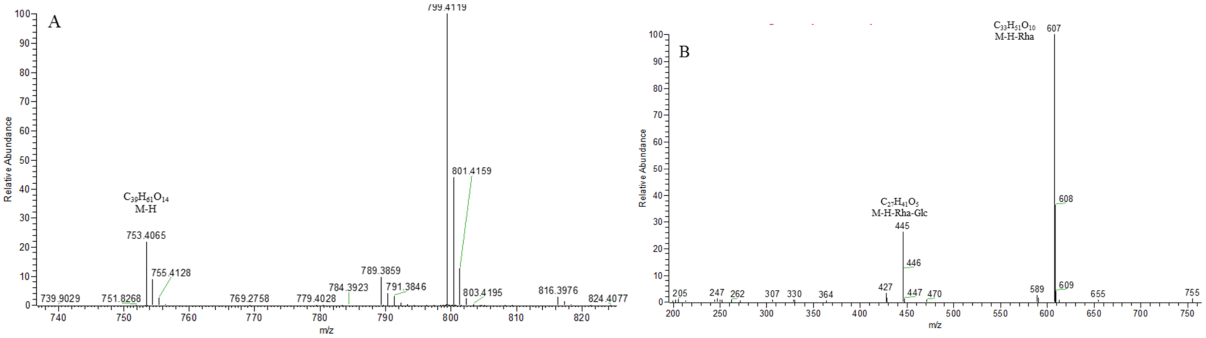

Figure S2. The ESI-MS spectrum (A) and ESI-MS/MS spectrum (B) of Ophiogenin 3-O-α-L-rha-(1→2)-β-D-glc in negative ion

mode.

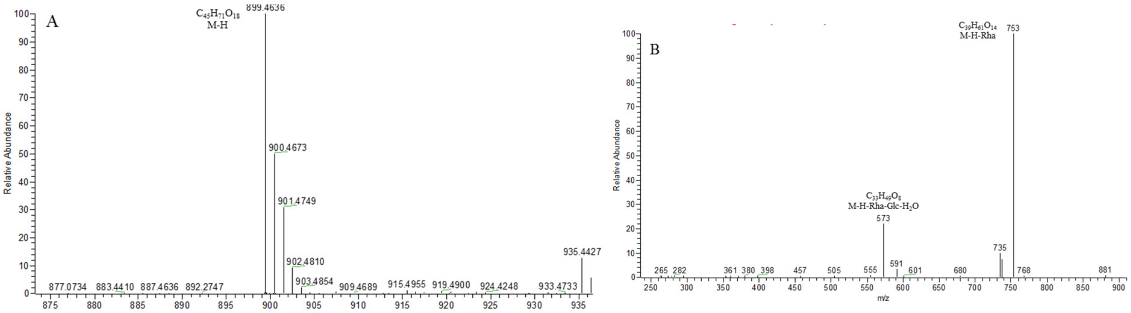

Figure S3. The ESI-MS spectrum (A) and ESI-MS/MS spectrum (B) of

(20R,25R)-26-O-β-D-glc-3β,26-dihydroxycholest-5-en-16,22-dioxo-3-O-α-L-rha(1→2)-β-D-Glc in negative ion mode.

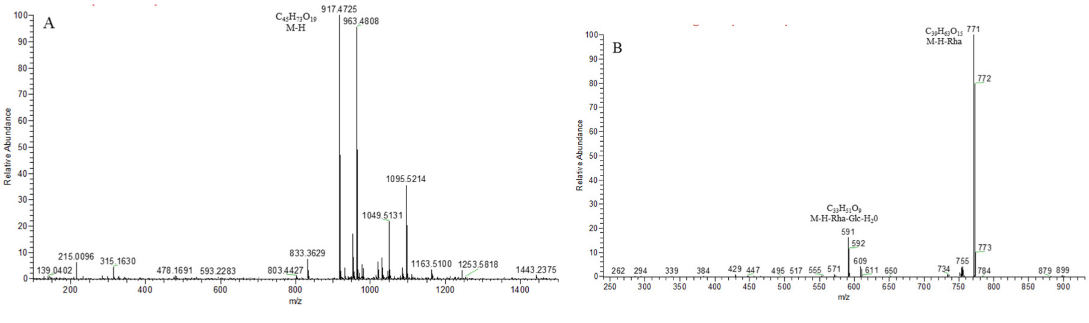

Figure S4. The ESI-MS spectrum (A) and ESI-MS/MS spectrum (B) of Ophiofurspiside M in negative ion mode.

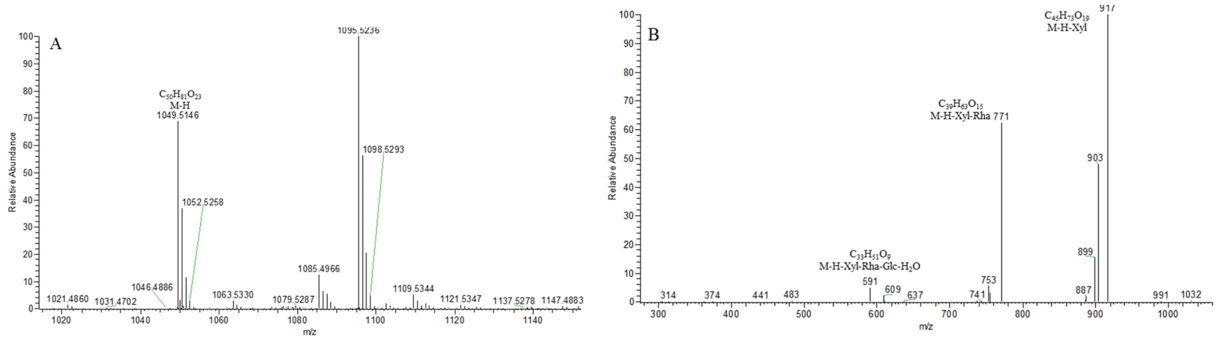

Figure S5. The ESI-MS spectrum (A) and ESI-MS/MS spectrum (B) of Xyl-Ophiofurspiside M in negative ion mode.

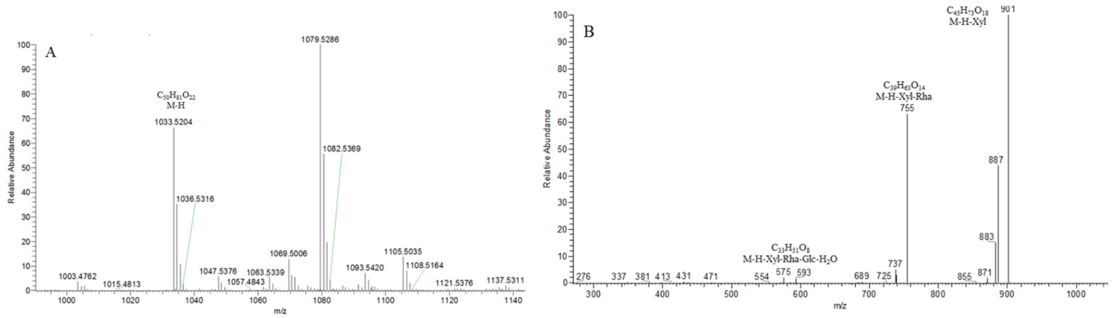

Figure S6. The ESI-MS spectrum (A) and ESI-MS/MS spectrum (B) of Ophiofurspiside A in negative ion mode.

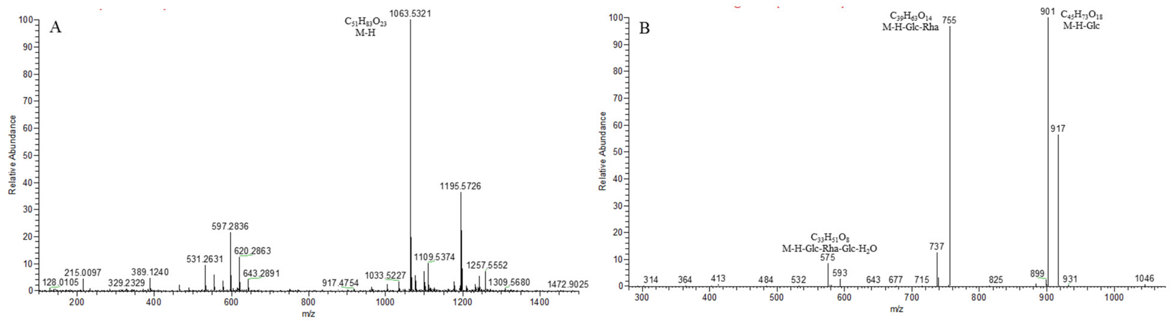

Figure S7. The ESI-MS spectrum (A) and ESI-MS/MS spectrum (B) of Ophiopogonin H in negative ion mode.

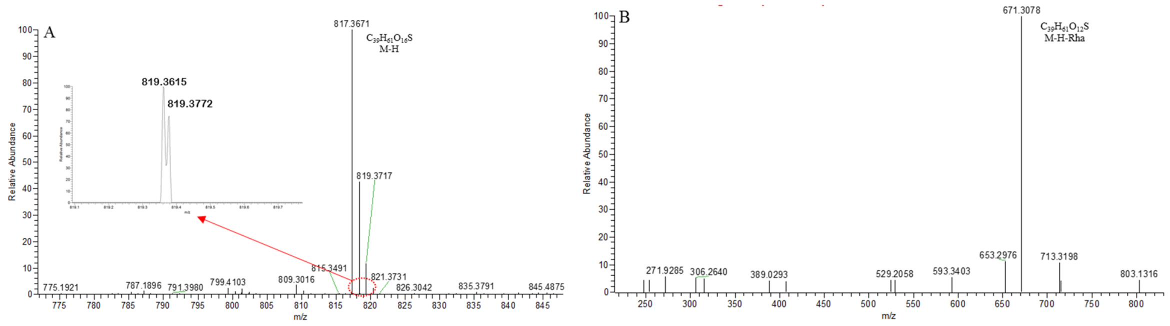

Figure S8. The ESI-MS spectrum (A) and ESI-MS/MS spectrum (B) of SS10-SS14 in negative ion mode.

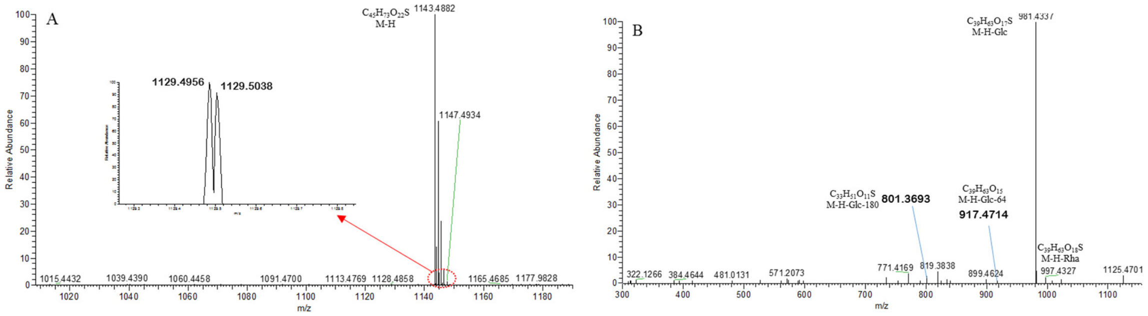

Figure S9. The ESI-MS spectrum (A) and ESI-MS/MS spectrum (B) of SS72-SS74 in negative ion mode.
